# miR-494-3p is a novel tumor driver of lung carcinogenesis

**DOI:** 10.18632/oncotarget.13933

**Published:** 2016-12-14

**Authors:** Alice Faversani, Stefano Amatori, Claudia Augello, Federico Colombo, Laura Porretti, Mirco Fanelli, Stefano Ferrero, Alessandro Palleschi, Pier Giuseppe Pelicci, Elena Belloni, Giulia Ercoli, Anna Degrassi, Marco Baccarin, Dario C. Altieri, Valentina Vaira, Silvano Bosari

**Affiliations:** ^1^ Division of Pathology, Fondazione IRCCS Ca Granda Ospedale Maggiore Policlinico , Milan, Italy; ^2^ Department of Biomolecular Sciences, University of Urbino Carlo Bo, Molecular Pathology Lab. PaoLa, Fano, Italy; ^3^ Department of Pathophysiology and Transplantation, University of Milan, Milan, Italy; ^4^ Flow Cytometry Service, Fondazione IRCCS Ca Granda, Ospedale Maggiore Policlinico, Milan, Italy; ^5^ Department of Biomedical, Surgical and Dental Sciences, University of Milan, Milan, Italy; ^6^ Division of Thoracic Surgery and Lung Transplantation, Fondazione IRCCS Ca Granda Ospedale Maggiore Policlinico, Milan, Italy; ^7^ Department of Experimental Oncology, European Institute of Oncology, Milan, Italy; ^8^ Department of Oncology and Hemato-oncology, University of Milan, Milan, Italy; ^9^ Biology Department, Nerviano Medical Sciences s.r.l., Nerviano, Italy; ^10^ Laboratory of Medical Genetics, Fondazione IRCCS Ca Granda Ospedale Maggiore Policlinico, Milan, Italy; ^11^ Prostate Cancer Discovery and Development Program, Tumor Microenvironment and Metastasis Program, The Wistar Institute, Philadelphia, Pennsylvania, United States of America

**Keywords:** miR-494-3p, miRNA, NSCLC, NOTCH1, stem cells

## Abstract

Lung cancer is the leading cause of tumor-related death worldwide and more efforts are needed to elucidate lung carcinogenesis. Here we investigated the expression of 641 miRNAs in lung tumorigenesis in a K-Ras^(+/LSLG12Vgeo);RERTn(ert/ert)^ mouse model and 113 human tumors. The conserved miRNA cluster on chromosome 12qF1 was significantly and progressively upregulated during murine lung carcinogenesis. In particular, miR-494-3p expression was correlated with lung cancer progression in mice and with worse survival in lung cancer patients. Mechanistically ectopic expression of miR-494-3p in A549 lung cancer cells boosted the tumor-initiating population enhanced cancer cell motility, and increased the expression of stem cell-related genes. Importantly, miR-494-3p improved the ability of A549 cells to grow and metastasize *in vivo*, modulating NOTCH1 and PTEN/PI3K/AKT signaling.

Overall, these data identify miR-494-3p as a key factor in lung cancer onset and progression and possible therapeutic target.

## INTRODUCTION

Despite an improved understanding of lung carcinogenesis, lung cancer is still a major cause of cancer-related death worldwide with increasing incidence in non-smoker subjects. Non Small Cell Lung Cancers (NSCLCs) comprise the majority of cases and genetic aberrations in cancer nodal genes, such as TP53, kRAS, EGFR, and ALK among others, have been recently described. Although much effort is focused in early disease detection through the introduction of novel biomarkers, the prognosis of NSCLC patients is poor and the rate of recurrence is unacceptably high [1]. Genetic models of disease represent a valuable tool to gain insights into molecular aberrations that underpin tumor onset and progression [2]. Genetically engineered mouse models may also uncover pre-neoplastic precursor lesions, which are often absent in human samples. Therefore, in this investigation, we studied the conditional knock-in K-Ras^(+/LSLG12Vgeo);RERTn(ert/ert)^ mouse model of lung cancer [3] and systematically profiled microRNA (miRNA) contents from histologically defined normal, hyperplastic, adenomatous and tumor lung specimens. In this model, lung carcinogenesis is driven by oncogenic K-Ras^G12V^ induction by 4-hydroxitamoxifen (4-OHT) administration [4]. Lung adenocarcinoma arises with complete penetrance, evolving from normal to hyperplasia, adenoma and finally cancer [3, 5].

Different studies highlighted miRNAs involvement in human tumors, including NSCLCs [6], and implicated their roles as oncogenes [7] or tumor suppressors [2, 8] through post-transcriptional modulation of gene expression. Several molecular and epigenetic mechanisms are involved in miRNAs deregulation in cancer, mostly because miRNAs are often localized at fragile sites [9], cancer susceptibility loci [9] or in proximity of CpG islands [10]. Despite numerous studies on miRNA deregulation in lung cancer, a consensus on which miRNAs are crucial for lung carcinogenesis and progression has remained elusive.

In this study, we aimed to identify mechanisms of disease conserved between experimental models of disease and NSCLC patients to eventually provide novel theranostics for early lung cancer diagnosis and, potentially, for novel therapeutic strategies.

## RESULTS

### Chromosome 12qF1 miRNAs expression is increased during lung tumorigenesis

**Figure 1:**
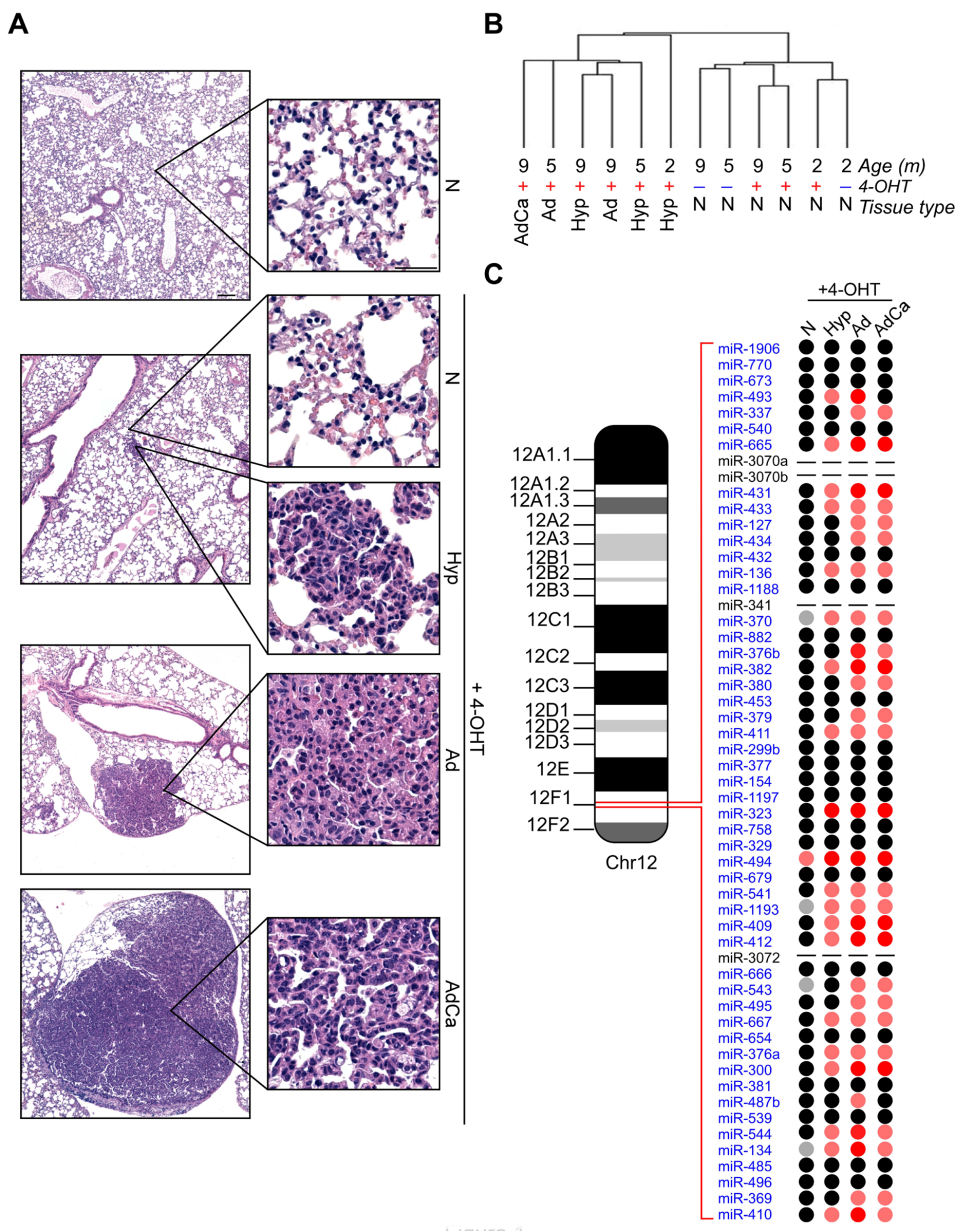
Overexpression of chr.12qF1 miRNAs during lung carcinogenesis in the K-Ras **^(+/LSLG12Vgeo);RERTn(ert/ert)^** mouse model. **A**. Representative images of mice lung tumorigenesis: normal lung tissue (N) without Tamoxifen administration; non-neoplastic lung tissue after Tamoxifen administration (4-OHT+ N); hyperplasia (Hyp); lung adenoma (Ad); lung adenocarcinoma (AdCa). Scale bar 100 µm or 50 µm (insets). **B**. Unsupervised hierarchical clustering of microdissected murine tissues profiled for miRNAs contents. **C**. Schematic representation of chr.12qF1 miRNAs and their expression levels during murine lung cancer progression. miRNAs Relative Quantities (RQ) were calculated over normal samples, without K-Ras^v12^ oncogene induction by 4-OHT administration. Grey spot: RQ ≤ 0.2; Pink spot:5≤RQ≤50; Red spot: RQ≥50; Black spot: no expression variation; − : miRNA not present within the TaqMan Low Density Array.

We profiled 641 miRNAs in laser-assisted microdissected samples from normal Tamoxifen non-induced (N), or Tamoxifen induced non-neoplastic (4-OHT+ N) lung parenchyma and neoplastic tissues (Hyperplasia, Hyp, Adenoma, Ad and Adenocarcinoma, AdCa) isolated from K-Ras^(+/LSLG12Vgeo);RERTn(ert/ert)^ mice, a well-established model of lung cancer tumorigenesis (Figure 1A; [3]). This analysis showed extensive miRNAs deregulation during lung cancer development (Supplementary Tables 1-6 and Supplementary Figure 1A). In particular, non-neoplastic tissues exhibited a distinctive miRNAs expression profiling compared to pre-neoplastic and cancerous lesions (Figure 1B). Interestingly, most of the miRNAs belonging to the murine chromosome 12qF1 cluster (30 out of 54 miRNAs, 55%) were overexpressed during oncogenic lung transformation (Figure 1C). Single qPCR analyses confirmed the upregulation of chromosome 12qF1 miRNAs (Supplementary Figure 1B).

Since 12qF1 miRNAs expression has been correlated with mice aging [11, 12], we next analyzed miRNAs levels in non-neoplastic lung tissues from K-Ras^(+/LSLG12Vgeo);RERTn(ert/ert)^ mice at different ages. Our data show that 12qF1 miRNAs were not affected by lung aging, as 26 miRNAs (48%) were not expressed in all samples and 15 miRNAs (28%) exhibited comparable levels in all specimens. Indeed, only nine (17%) and 4 (7%) miRNAs were decreased or overexpressed (2.5-folds difference), respectively, in older tissues (Supplementary Figure 1C). Therefore, the 12qF1 miRNAs upregulation in lung tissues from the K-Ras^(+/LSLG12Vgeo);RERTn(ert/ert)^ mouse model is correlated with lung carcinogenesis.

### The 14q32 miR-494-3p is correlated to lung cancer patients’ prognosis

**Figure 2:**
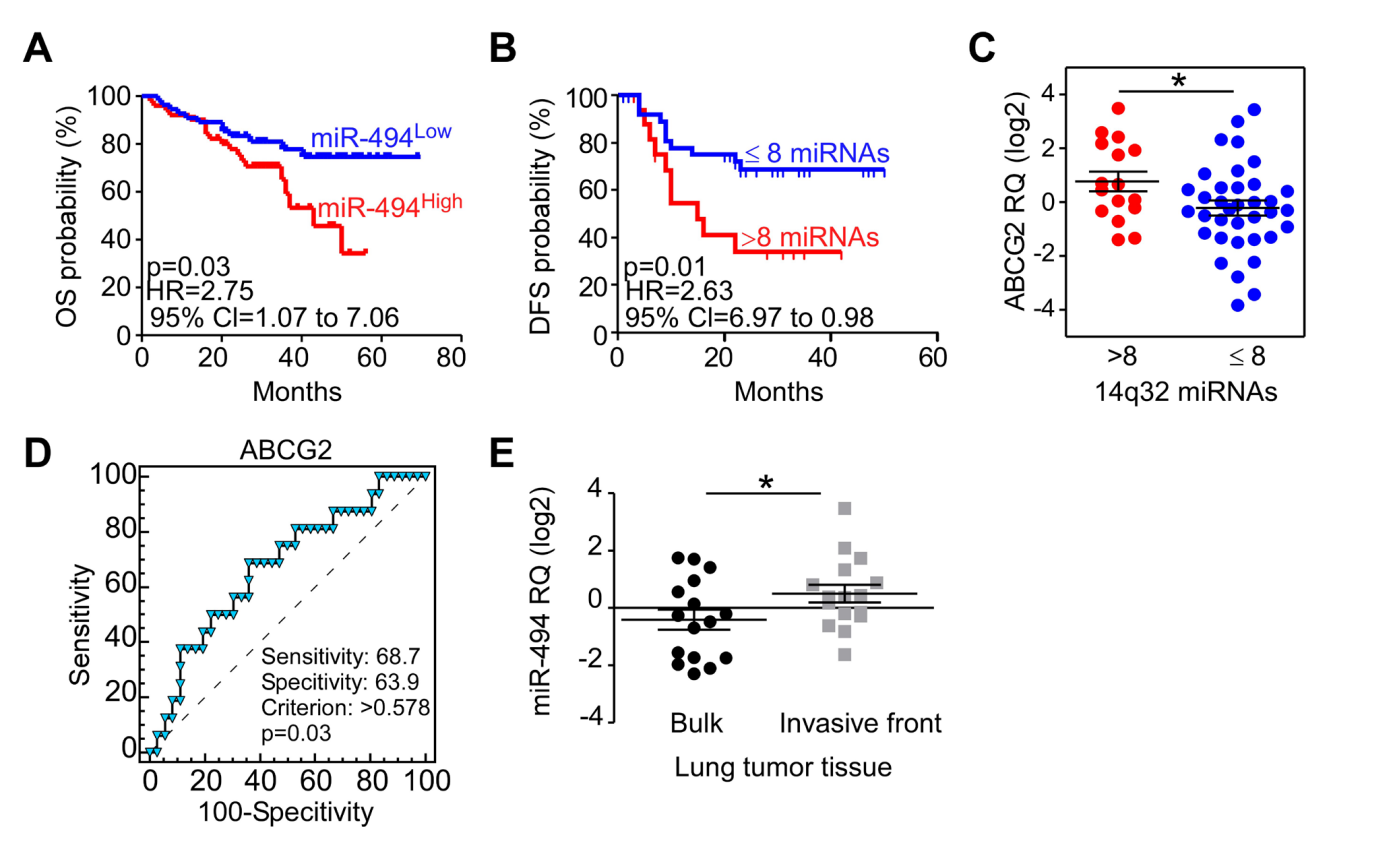
Chromosome 14q32 miRNAs and NSCLC progression in humans **A**. Kaplan-Meier curves of NSCLC patients overall survival according to miR-494-3p levels. *p* = 0.03 by Log-Rank test. **B**. Kaplan-Meier curve of NSCLC patients’ disease-free survival according to expression of chr.14q32 miRNAs. *p* = 0.01 by Log-Rank test. HR, Hazard Ratio; CI, Confident Interval. **C**. ABCG2 gene expression was analyzed in NSCLC tissues of patients with (>8) or without (≤8) major upregulation of 14q32 miRNAs.*, *p* = 0.04 by unpaired *t* test. **D**. ROC analysis was performed to identify the ability of ABCG2 to discriminate between high and low chr.14q32 miRNAs expressors (*p* = 0.03). **E**. miR-494-3p analysis in tumor bulk and invasive front of 16 NSCLC cases. *, *p* = 0.03 by paired *t* test.

12qF1 murine miRNAs region is conserved in the human genome and maps to the DLK1-DIO3 locus on chromosome 14q32. We therefore analyzed a subset (*n* = 10) of those miRNAs (miR-127, -300, 370, -379, -382, -409-3p, -412, -431, -494-3p and -543) in a series of 57 NSCLC patients. Although these miRNAs were not generally significantly overexpressed in lung cancer compared to normal tissues (Supplementary Figure 2A), patients with high levels of miR-494-3p had a shorter disease-free survival time (*p* = 0.046; Supplementary Figure 2B). To further substantiate these findings, we next examined a larger cohort of 113 NSCLC patients. High miR-494-3p levels were significantly associated with shorter patients’ overall survival (*p* = 0.03; Figure 2A). No other miRNA individually correlated with NSCLC patients’ survival. However, the elevated presence of the majority of 14q32 miRNAs (9 out of 10 miRNAs) in tumor tissues was associated with shortened disease-free survival (*p* = 0.01; Figure 2B).

Chromosome 14q32 miRNAs expression has been previously associated with a stem-like subtype in hepatocellular carcinoma [13]. Accordingly, we next looked at a potential relationship between 14q32 miRNAs expression and cancer stem cell markers, and we examined the expression of *CD44*, *ABCG2*, *ALDH1A1*, *NANOG* and *c-MYC* genes in 53 tumor samples (Figure 2C and Supplemental Figure 2C). *ABCG2* was increased in tumors with concurrent chromosome 14q32 miRNAs upregulation (Figure 2C). Moreover, ROC analyses demonstrated that *ABCG2* levels accurately discriminated NSCLC patients with high- or low-miRNAs expressors (Figure 2D; *p* = 0.03).

To begin to test a role of this association in disease dissemination [14], we next examined the expression of 14q32 miRNAs in 16 cases of NSCLC where tumor bulk and the tumor invasive front were isolated by laser-assisted microdissection. miR-494-3p expression was significantly higher in the invasive front than in tumor bulk specimens (*p* = 0.03; Figure 2E). The expression of other miRNAs was indistinguishable between the two tumor areas (Supplementary Figure 2D).

Together, the results obtained with the murine lung cancer model and analysis of patient cohorts suggested that miR-494-3p and, to a lesser extent, chromosome 14 miRNA cluster correlated with a more invasive, *ABCG2*-positive phenotype in NSCLC.

### Regulation of miR-494-3p expression

**Figure 3:**
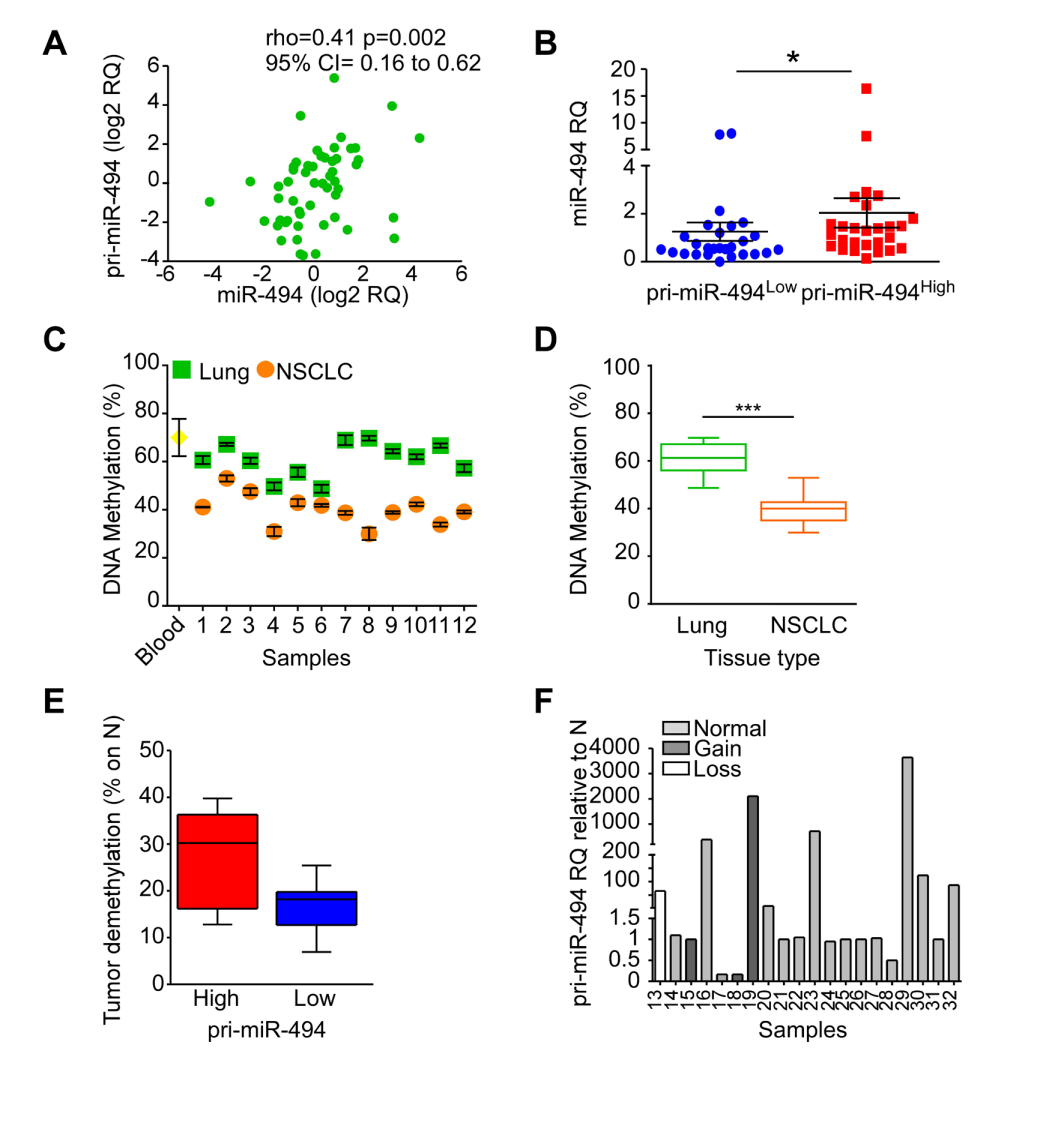
Regulation of miR-494-3p expression **A**., **B**. Pri-miR-494 expression was investigated in 57 NSCLC samples and compared with miR-494-3p levels. A) miR-494-3p and pri-miR-494 expression correlation. Spearman's rank correlation and coefficient rho along with 95% confidence intervals (CI) are shown (*p* = 0.002). Each point represents a sample. B) Pri-miR-494 expression levels compared with miR-494-3p levels. *, *p* = 0.02 by unpaired *t* test. Each point represents a sample. **C**., **D**. Methylation status of 16 CpG sites at the IG-DMR of human DLK1-DIO3 region was analyzed in 12 matched lung normal and NSCLC specimens as well as in a control sample (peripheral blood from healthy individual). Significant reduction of methylation was found in tumor samples compared to their normal counterpart. ***, *p* = 0.0005 by Mann-Whitney U test. **E**. The IG-DMR decreased methylation of NSCLC samples (*n* = 12) was analyzed in function of pri-miR-494 expression levels. **F**. Chromosome 14q genomic alterations were analyzed by a-CGH in 20 tumor samples. Individual pri-miR-494 levels are displayed in NSCLCs according to the genomic alteration found. Bar, individual sample.

The 14q32 locus undergoes imprinting [15], and we next further characterized how miR-494-3p expression is regulated in human lung tissues. Firstly, we demonstrated in our series of 57 NSCLCs that there is a direct correlation between the precursor pri-miR-494 levels and its mature miR-494-3p expression (*p* = 0.002; Figure 3A). In addition, tumors with high pri-miR-494 expression showed higher miR-494-3p levels (*p* = 0.02; Figure 3B).

We next looked at the epigenetic status of the 16 CpG sites at the intergenic differentially methylated (IG-DMR) region, which contributes to the imprinting of the DLK1-DIO3 locus [15], in a subset of matched normal lung and cancer tissues. Normal tissues showed a significant higher IG-DMR CpG methylation levels (mean: 60.9%) than tumor (mean: 40%) counterparts (*n* = 12; Figure 3C, 3D; *p* = 0.0005). In addition, IG-DMR demethylation in tumors relative to non-neoplastic lung parenchyma was more evident in NSCLCs with elevated pri-miR-494 levels (Figure 3E). Finally, we examined submicroscopic genomic alterations at the q11.2-q32.33 region of chromosome 14 by a-CGH in subsets of lung tissues and paired non-neoplastic counterparts (*n* = 20). This analysis showed that the 14q region was marginally affected by genomic rearrangements, since it was gained in 4 samples (20%), lost in one specimen (5%), and unchanged in 15 samples (75%) (Supplementary Table 7). Despite sample 17 showed a gain at q32.32-q32.33 band, this region does not include the DLK1-DIO3 locus (q32.2-q32.31). Further, there was no direct correlation between 14q genomic status and pri-miR-494 expression (Figure 3F). These data show that epigenetic but not genetic alterations could contribute to miR-494-3p deregulation in lung cancer, prompting us to investigate potential miR-494-3p-directed signaling pathways in NSCLC.

### miR-494-3p regulates pathways involved in cancer and development

**Figure 4:**
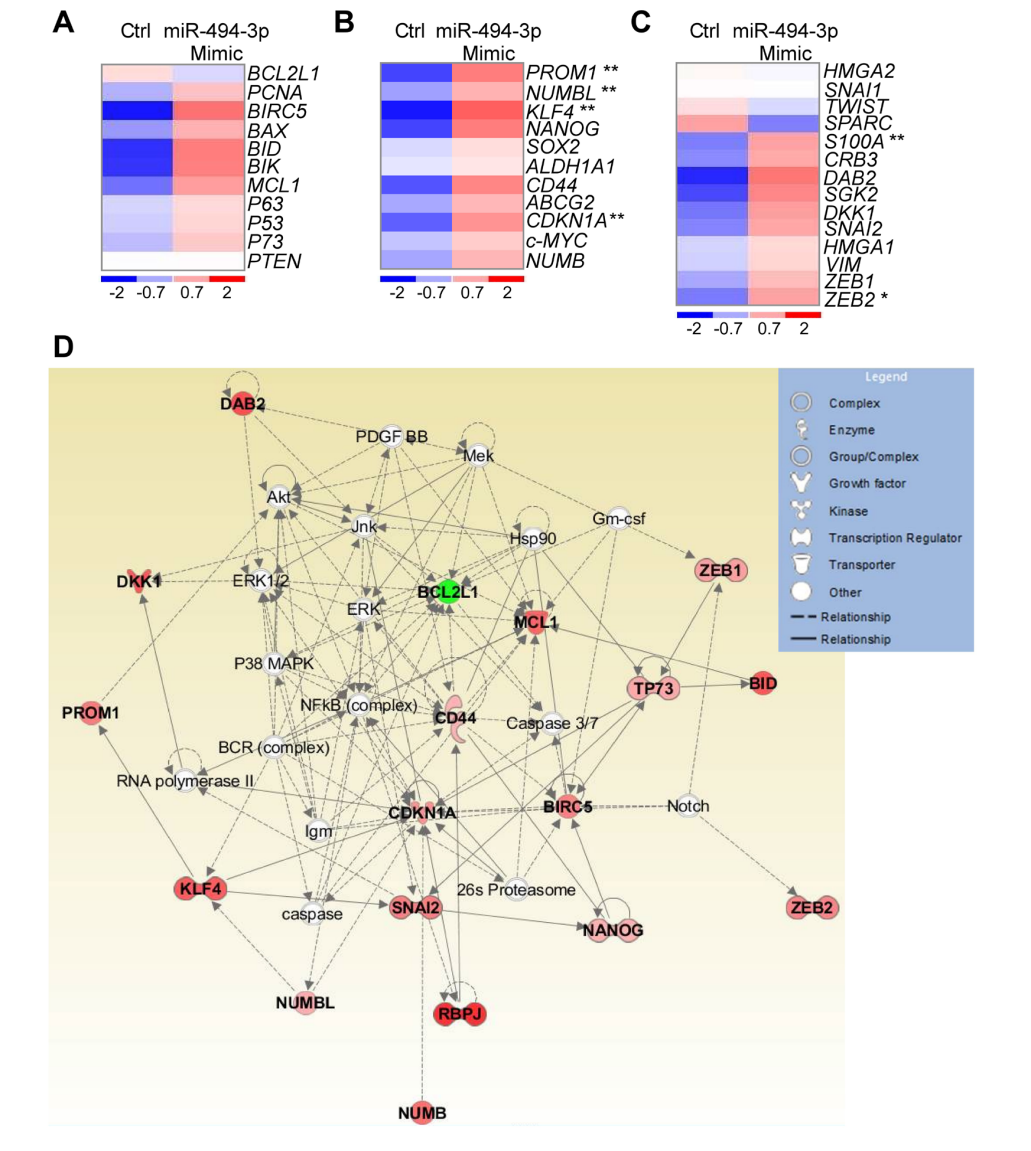
miR-494-3p involvement in molecular pathways relevant for cancer progression **A**.-**C**. Heat-Maps of genes involved in apoptosis (A), cancer stem cells maintenance (B; *PROM1*, *p* = 0.007; *NUMBL*, *p* = 0.001; *KLF4*, *p* = 0.005; *CDKN1A*, *p* = 0.003, by Mann-Whitney U test), or epithelial-to-mesenchymal transition (C; *ZEB2*, *p* = 0.03; *S100A*, *p* = 0.003, by Mann-Whitney U test) upon enforced expression of a control (Ctrl) or miR-494-3p Mimic in A549 cultures. One experiment representative of four is shown. Red and blue colors indicate over or underexpression of the gene in miR-494-3p-expressing cells, respectively. **D**. Ingenuity pathway analysis of the 36 analyzed genes in A549 cells with ectopic miR-494-3p or control construct expression returned a signaling network with impact on cell survival, embryonic and organism development (Score 45). Genes found to be overexpressed or downmodulated in A549 cells transfected with the miR-494-3p compared to the control are shown respectively in red and green.

For these studies, we first analyzed the expression of 36 genes implicated in key cancer pathways (Supplementary Table 8) after modulation of miR-494-3p expression in A549 cells (Supplementary Figure 3A). Interestingly, forced expression of miR-494-3p did not affect apoptosis-related mechanisms (Figure 4A), but significantly increased the levels of *PROM1*, *CDKN1A*, *NUMBL* and *KLF4*, four stem cell-related mRNAs, compared to controls (*p* = 0.007, 0.003, 0.001 and 0.005, respectively; Figure 4B). Consistent with these findings [16], factors involved in epithelial-to-mesenchymal transition (EMT) were overexpressed after upregulation of miR-494-3p (Figure 4C). In particular, *ZEB2* and *S100A* mRNAs were significantly increased upon miR-494-3p overexpression (*p* = 0.03 and 0.003, respectively; Figure 4C). Ingenuity pathway analysis suggested that miR-494-3p modulation influenced cellular networks implicated in “cell death and survival, embryonic development and organism development” (Score 45; focus molecules: 18; Figure 4D). Based on these findings, we next looked at a potential role of miR-494-3p in potentially modulating lung cancer “stemness”.

### miR-494-3p is involved in lung tumor-initiating population maintenance

**Figure 5:**
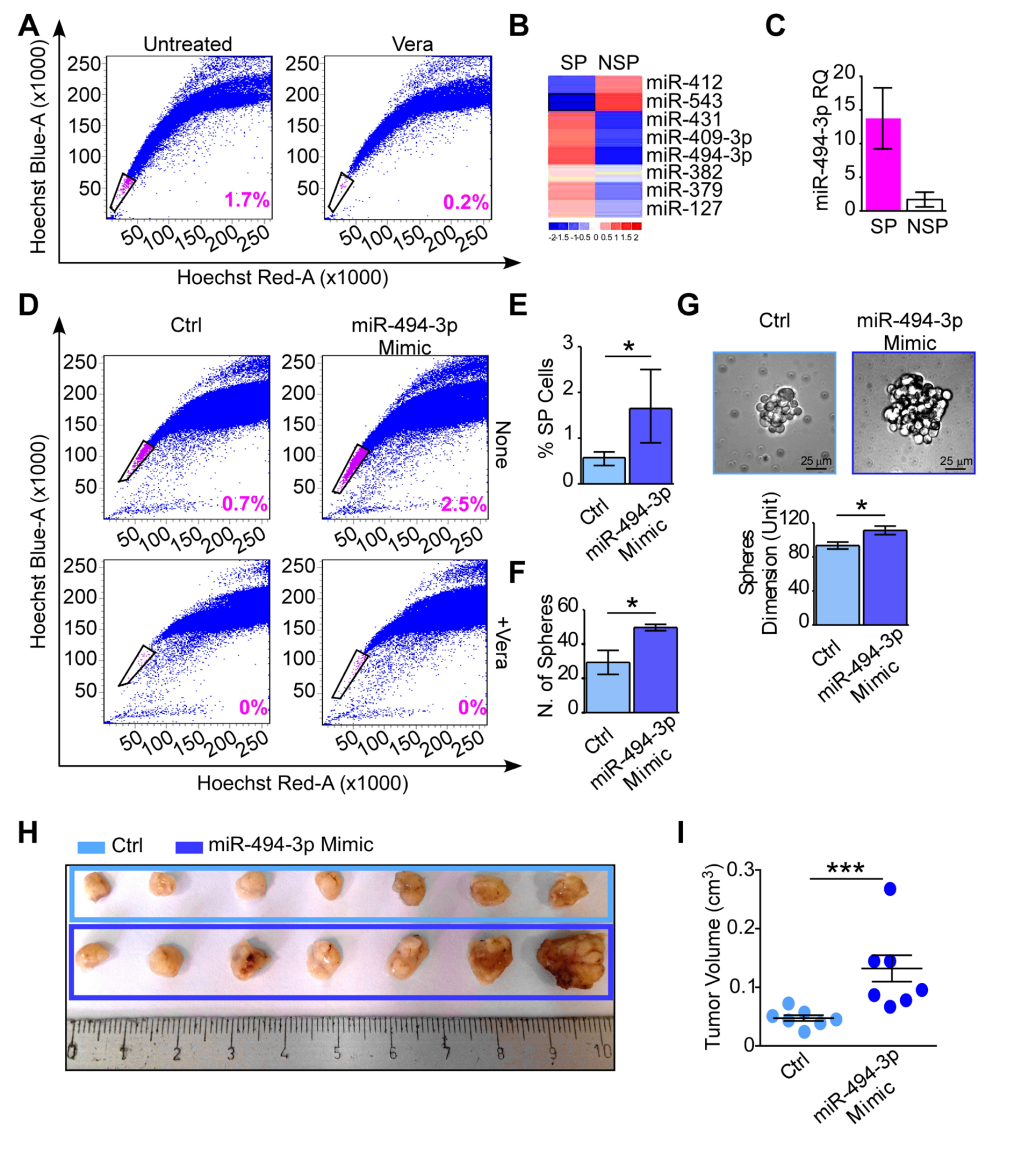
miR-494-3p contributes in cancer progenitor cells maintenance and tumor growth ***in vivo***. **A**. The side population (SP) analysis was performed in A549 cultures and the SP and nonSP (NSP) were isolated as the population that disappears after Verapamil incubation (+Vera) by FACS sorting. The SP percentage is shown. **B**. Chromosome 14q32 miRNAs expression was investigated in SP and NSP components. The heatmap shows miRNAs expression levels in the two populations. Red and blue colors indicate high or low expression, respectively. **C**. miR-494-3p expression in A549-SP and -NSP populations (fold difference = 8.2). Bars represent the average miR-494-3p expression level (RQ) from three independent experiments. **D**., **E**. A549 cells were transfected with a miR-494-3p or a control (Ctrl) Mimic and the SP was analyzed by FACS as in A. The percentage of the SP is shown within each graph and quantified from four independent experiments in E. *, *p* = 0.02 by Mann-Whitney U test. **F**., **G**. A549 cells with forced miR-494-3p or a control Mimic expression were plated in low-adhesion plates in serum-free media. The ability of A549 cells to form spheres was investigated after 7 days when the number (F) and size (G) of A549-spheres were analyzed (*, *p* = 0.04 for both analyses, by Mann-Whitney U test). Representative images of A549-sphere are shown (G). Scale bars indicate 25 µm. Bars represent the average from three independent experiments. **H**., **I**. A549 cultures transfected with a miR-494-3p or a control (Ctrl) Mimic were subcutaneously injected in the right flank of athymic nude mice (*n* = 7 per condition). Tumor growth was monitored with a caliper for 18 days, after which mice were sacrificed and tumors were harvested (H). Tumor growth (I) was calculated using the formula: Volume = (width)^2^ × length/2. ***, *p* = 0.006 by Mann-Whitney U test.

To better understand a potential involvement of 14q32 miRNAs and, in particular, miR-494-3p in tumor-initiating progenitor maintenance, the side (SP) and non-side (NSP) cell populations were sorted from A549 lung cancer cells (Figure 5A). Eight out of the ten analyzed 14q32 miRNAs were expressed by A549 cells at baseline conditions (namely miR-127,-379, -382, -409-3p, -412, -431, -494-3p and -543; Supplementary Figure 3B). Importantly, SP cells exhibited a distinctive miRNAs expression profile compared to NSP cells (Figure 5B), as miR-494-3p miRNA was the most overexpressed miRNA in the SP fraction (FC = 8.2; Figure 5C).

Next, we overexpressed miR-494-3p in A549 and examined the percentage of SP cells compartment by FACS. Higher levels of miR-494-3p resulted in a significant increase of SP, compared to controls (*p* = 0.02; Figure 5D-5E). Conversely, reduction of miR-494-3p levels in A549 by transfection with a miRNA Inhibitor (*p* = 0.03; Supplementary Figure 3C) did not affect the SP compartment compared to control (Supplementary Figure 3D). Ectopic miR-494-3p expression in non-adherent culture condition significantly enhanced A549 sphere formation ability and size, a marker of stemness, compared to control incubations (*p* = 0.04 for both analyses; Figure 5F, 5G). To further test a role of miR-494-3p in sustaining tumor growth *in vivo*, A549 cells overexpressing miR-494-3p or control miRNA were injected subcutaneously in the lower flank of athymic mice (*n* = 14; Figure 5H). A549 cells with forced overexpression of miR-494-3p generated larger tumors compared to control cultures 18 days after injection (*p* = 0.006; Figure 5I).

### miR-494-3p overexpression enhances tumor motility *in vitro* and *in vivo*

**Figure 6:**
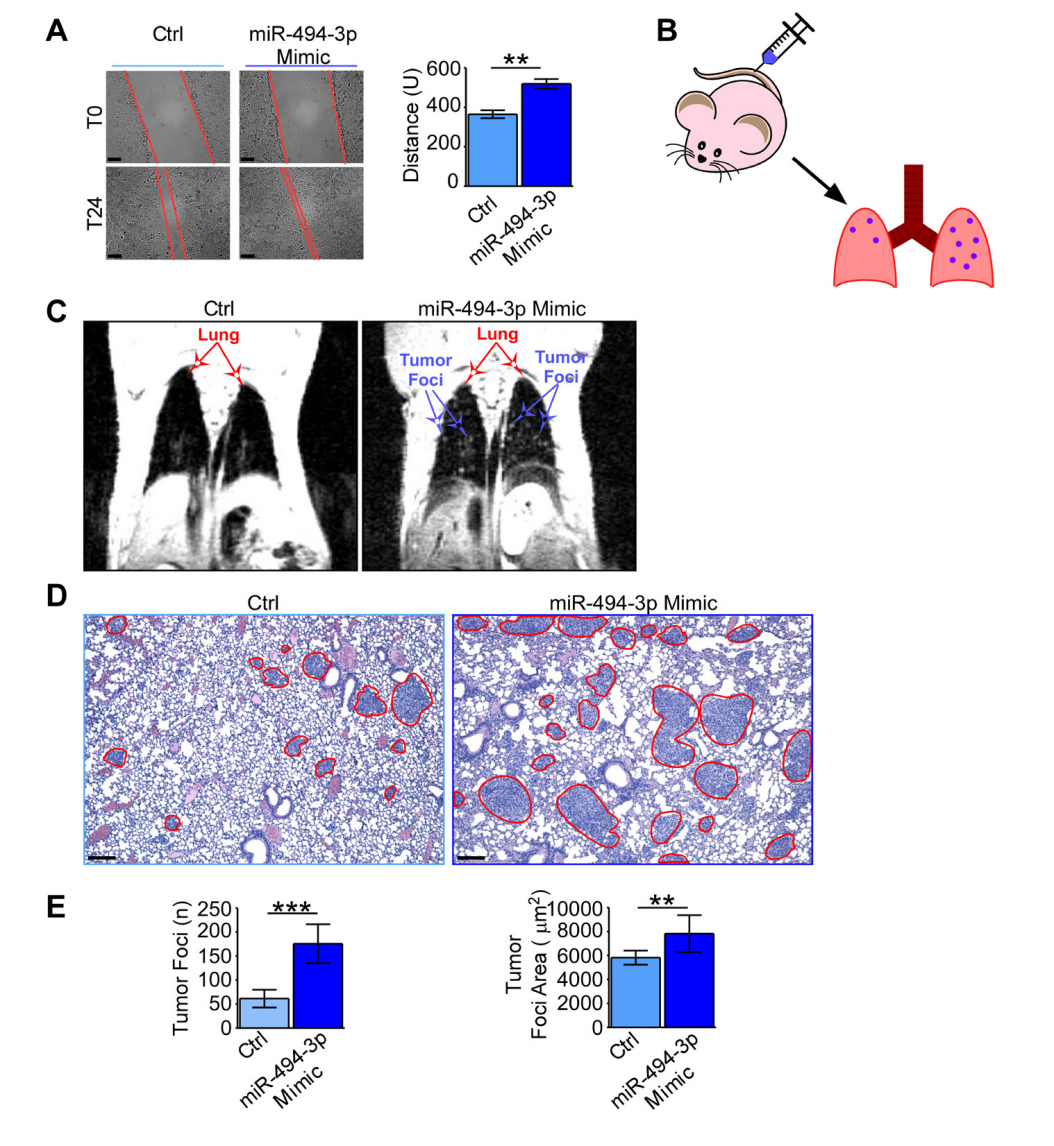
miR-494-3p enhanced lung cancer cells motility ***in vitro*** and ***in vivo***. **A**. A549 cells were transfected with a miR-494-3p or a control (Ctrl) Mimic. 48h from transfection (T0) a wound was made in the monolayer and cells were allowed to migrate for 24h (T24). Representative images at T0 and T24 are shown (*left*). Scale bars, 100 µm. *Right*, quantification of the wound closure. Bars represent the average from three independent experiments, U, arbitrary units; **, *p* = 0.008 by unpaired Student's *t* test. **B**.-**E**. A549 cultures transfected with a miR-494-3p or a control Mimic were intravenously injected into the lateral tail vein of athymic nude mice (*n* = 13 per condition; B). The onset of lung tumor metastasis was monitored using MRI (C) and mice were sacrificed after 18 days from the injection. At harvesting, lungs were FFPE (D) and the number and area of metastatic foci (red lines) were identified and scored on H&E sections (scale bars, 100 µm). A549 cells transfected with miR-494-3p generated significantly more and larger metastatic foci than controls (E). ***, *p* = 0.0002 and **, *p* = 0.002 by Mann-Whitney U test.

Based on the above results, we next analyzed the role of miR-494-3p in sustaining tumor cell motility and metastatic dissemination *in vivo*. In a wound-healing assay, overexpression of miR-494-3p enhanced A549 cells migration compared to control samples (Ctrl) 72 h after transfection (*p* = 0.008; Figure 6A). Moreover, we confirmed the ability of miR-494-3p to enhance cell migration by a Boyden chamber assay (*p* = 0.02; Supplementary Figure 4A and B).

Then, A549 cells overexpressing miR-494-3p or a control miRNA (Ctrl) were injected in the lateral tail vein of athymic mice (*n* = 26, Figure 6B) and lung cancer foci formation was monitored by MRI at day 11 and 17 after injection (Figure 6C). After harvesting mice lungs (day 18), metastatic foci were scored on histological sections (Figure 6D). Mice injected with A549-miR-494-3p cells exhibited a significant increase in the number and surface area of metastatic foci compared to controls (*p* = 0.0002 and *p* = 0.002, respectively; Figure 6E). No liver metastases were detected in mice (data not shown).

### miR-494-3p contribution to NOTCH1 pathway and PTEN regulation

**Figure 7:**
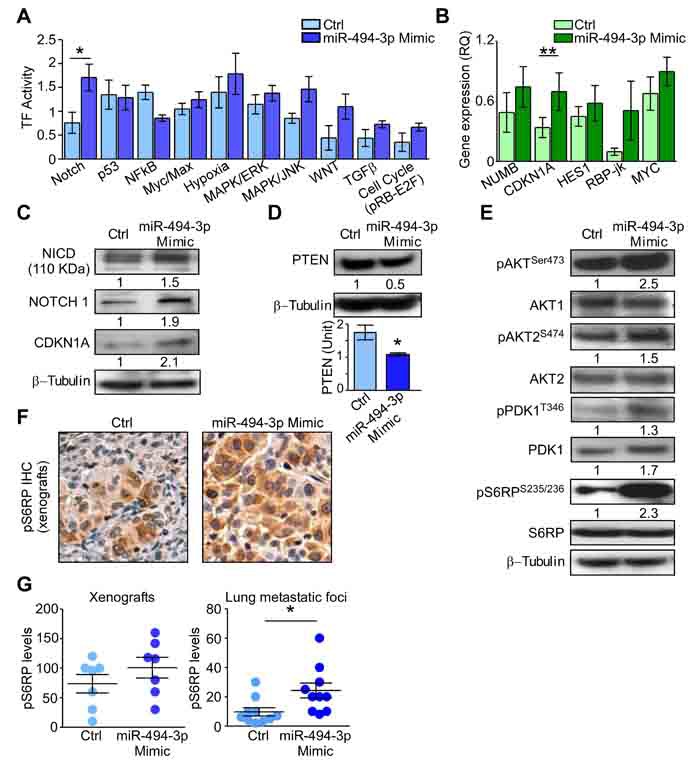
miR-494-3p contribution to NOTCH and PI3K/AKT pathways **A**. Quantification of transcription factor (TF) activity in A549 transfected with miR-494-3p or control (Ctrl) Mimic. Bars represent the average from six independent experiments. *, *p* = 0.04 by Mann-Whitney U test. **B**. The gene expression levels (RQ) of NOTCH1 targets was investigated in A549 transfected as in A. *CDKN1A* expression was significantly upregulated after miR-494-3p overexpression compared with control. Bars represent the average from three independent experiments. **, *p* = 0.003 by Mann-Whitney U test. **C**.-**D**. NOTCH1 and miR-494-3p targets protein contents were analyzed in A549 cultures overexpressing or not (Ctrl) the miRNA. Numbers indicate the quantification of proteins in miR-494-3p overexpressing cells compared to control. *, *p* = 0.04 by Mann-Whitney U test. **E**. Western Blot analysis of PI3K/AKT pathway members after miR-494-3p overexpression. Numbers indicate the quantification of proteins and phospho-proteins (normalized on the corresponding total protein) in miR-494-3p overexpressing cells compared to control. **F**. Xenografts generated from A549 cultures transfected with miR-494-3p or control (Ctrl) were analyzed for pospho-S6RP Ser235/236 by immunohistochemistry (pS6RP IHC). **G**. Immunostaining for phospho-S6RP Ser235/236 in A549-xenografts or in lung metastatic foci was scored multiplying the percentage of stained cells for the staining intensity. Tumors derived from A549 transfected with miR-494-3p Mimic showed higher pS6RP levels compared to controls. *, *p* = 0.01 by Mann-Whitney U test.

Finally, we examined signaling pathways downstream of miR-494-3p potentially implicated in tumor-initiating cells. Analysis of a reporter array of 10 oncogenic transcription factors (Supplementary Table 9, [17]), demonstrated that A549-miR-494-3p expressing cells exhibited significant increased NOTCH1 pathway activity (*p* = 0.04; Figure 7A). This was associated with significant upregulation of known NOTCH1 target genes, including *CDKN1A and RBP-jk* mRNAs (*p* = 0.003; Figure 7B). At the protein level, miR-494-3p upregulation increased both total NOTCH1 and NOTCH1 Intracellular Domain (NICD), as well as of CDKN1A (Figure 7C). Lastly, PTEN, a validated target of miR-494-3p [18, 19] and a NOTCH1-regulated protein [20], was significantly decreased in response to ectopic expression of miR-494-3p (*p* = 0.04; Figure 7D).

Based on these results of NOTCH1 activation and PTEN repression, we next looked at potential alterations of PI3K signaling in A549 cells with modulation of miR-494-3p expression. Consistent with the data above, miR-494-3p overexpression was associated with PI3K pathway activation, with increased levels of phosphorylated AKT at Ser473, AKT2 at Ser474, total and AKT-activated PDK1 (pPDK1^T346^), and phospo-S6RP protein (Figure 7E). In contrast, reduced levels of miR-494-3p did not affect NOTCH1 activation (Supplementary Figure 4C and D), the levels of the NOTCH1 target CDKN1A (Supplementary Figure 4E), or of PI3K signaling in A549 cells (Supplementary Figure 4F).

Similar findings were observed *in vivo*, as xenografts and metastatic foci generated by miR-494-3p-overexpressing A549 cells exhibited increased staining for phospo-S6RP (Figure 7F, 7G).

**Figure 8:**
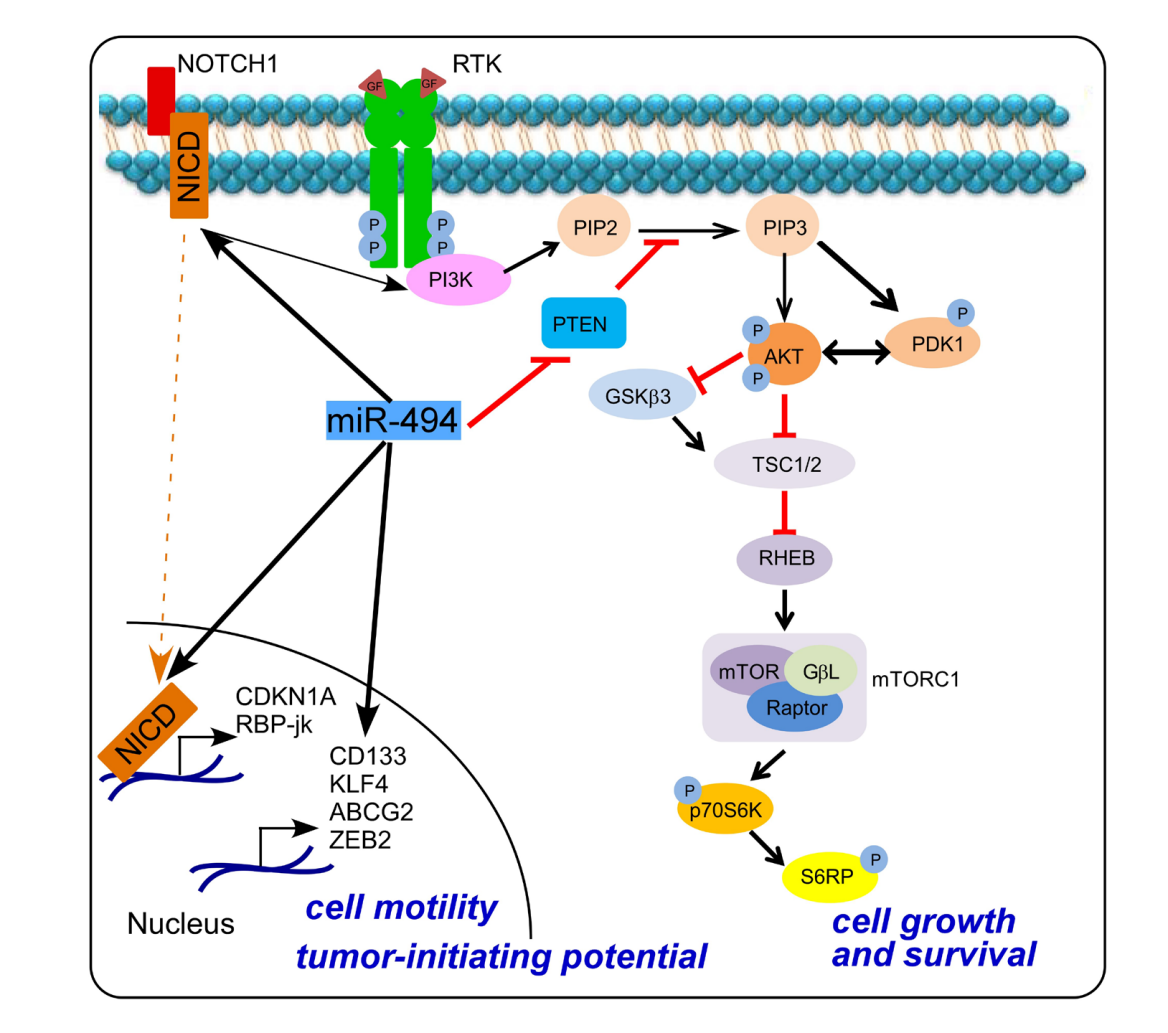
Schematic representation of miR-494-3p-NOTCH1-PI3K-AKT signaling in lung cancer cells

Therefore, our data provide comprehensive evidence that miR-494-3p promotes activation of PI3K signaling, with concomitant activation of AKT-mTOR-pS6RP in lung cancer, contributing to tumor cell proliferation. In parallel, miR-494-3p promotes NOTCH1 signaling and expression of downstream genes involved in EMT and cancer stemness (Figure 8). Together, these results point to a novel signaling axis orchestrated by miR-494-3p-NOTCH1-PI3K activation (Figure 8) involved in lung cancer onset and metastatic dissemination.

## DISCUSSION

Several studies demonstrated the importance of miRNAs in the progression of different types of human tumors [6, 21]. Accordingly, miRNAs have been implicated in key hallmarks of cancer, dampening apoptosis, inducing cell proliferation, eliminating tumor suppressor pathways, and promoting angiogenesis, cell migration and invasion. For these properties, miRNAs have been proposed as diagnostic, prognostic and predictive biomarkers in cancer, and may provide novel therapeutic opportunities [21].

In this study, we showed that the overexpression of the murine chr.12qF1 miRNA cluster is involved in lung cancer development in a conditional knock-in mouse model of disease. This cluster is conserved in humans at chromosome 14q32, where its orthologue, the DIO3-DLK1 miRNA cluster, is epigenetically deregulated in human lung cancer. More importantly, its overexpression or the upregulation of its member miR-494-3p, predicts a poorer prognosis in lung cancer patients.

In human lung tumors, miR-494-3p is highly expressed at the invasive front and its forced expression in lung cancer cells induces a stem-like phenotype, with increased side population compartment, tumor sphere formation, heightened cell motility, and increased NOTCH1 signaling. In turn, this promotes tumor growth and metastatic dissemination *in vivo*, potentially reflecting a general pro-tumorigenic role of miR-494-3p by simultaneously downregulating the PTEN tumor-suppressor and increasing NOTCH1 and PI3K-AKT-S6RP signaling *in vitro* and *in vivo*.

Human chromosome 14q32 is an imprinted locus, which harbors both maternally (*MEG3*, *MEG8* and *asRTL1*) and paternally expressed genes (*DLK1*, *DIO3* and *RTL1*). This region contains 54 paternally imprinted miRNAs that are organized in two segments and separated by a cluster of putative C/D box small nucleolar RNAs [13]. The overexpression of miRNAs associated with chromosome 12qF1 has been demonstrated in different genetically engineered mouse models of carcinogenesis, such as the c-Met hepatocellular carcinoma model [13] and K-RasLSL-G12D lung cancer model [22]. Importantly, in these previous studies a strong correlation between 12qF1 miRNAs and cancer stem-cell features was observed [13, 22]. In human lung tumors, DIO3-DLK1 miRNAs have been correlated with poorer survival [23, 24] and with a cell motility gene signature [23]. Specifically, miR-494 has been shown to contribute to tumorigenesis of brain [25], lung [24] and liver [19] by affecting the PTEN/AKT pathway.

Our data validate an important role of miR-494-3p and DIO3-DLK1 miRNAs cluster as important biomarkers of lung carcinogenesis, highlighting that upregulation of those miRNAs is an oncogenic mechanism conserved in humans, where epigenetic deregulation of DIO3-DLK1 miRNAs is observed in more aggressive lung tumors. High levels of these miRNAs correlate with increased *ABCG2* gene expression in lung tumor specimens, a bona fide marker of stem cells and of the side population compartment [26]. Accordingly, DIO3-DLK1 miRNAs were highly expressed in the side population, stem cell-like compartment of lung cancer cells. Moreover, ectopic expression of the cluster member miR-494-3p enhanced the A549 side population compartment and *CD133*, *NUMBL* and *KLF4* mRNA levels.

With respect to disease outcome, we have shown here that miR-494-3p predicts shorter survival of NSCLC patients, and mechanistically promotes increased tumor growth and metastatic dissemination via activation of a NOTCH1-PI3K-AKT axis, a key signaling network of lung carcinogenesis and important therapeutic target [27]. NOTCH1 signaling is an evolutionarily conserved pathway which plays an important role in cell-fate determination, cell proliferation and survival [28]. Perturbation of NOTCH1 signaling has been shown in different solid tumors such as lung [29], breast [30], liver [31], gastric [32] and prostate [33] carcinomas and hematologic malignancies such as lymphomas, T-ALLs and CLLs [34], where it is associated with EMT and cancer stem cells self-renewal and preservation [35, 36]. Also, NOTCH1 pathway signaling is hyperactive during K-Ras^(+/LSLG12Vgeo);RERTn(ert/ert)^ mouse lung carcinogenesis and is required for tumor maintenance [37]. There is increasing evidence for an important link between the PI3K/AKT/mTOR pathway and NOTCH1 in sustaining the cancer stem cell niche [27]. In particular, NOTCH1 decreases PTEN protein levels through Hes1, a NOTCH1 transcriptional target, which binds *PTEN* promoter and represses its activity. Moreover, NOTCH1 increases Interleukin-7 Receptor α and Insulin-like Growth Factor 1 Receptor (IGF-1R) protein levels, which activate PI3K/Akt signaling. In lung adenocarcinoma, IGF-1R overexpression and Akt activation by NOTCH1 is stimulated by hypoxic microenvironment [38], and NOTCH1 targeting may provide a viable therapeutic approach for patients with K-Ras mutant lung adenocarcinoma [39].

In summary, we have identified miR-494-3p as a potentially critical theranostic marker of tumor progression in NSCLCs. These results may open concrete new prospects for targeting a NOTCH1-PI3K–AKT–mTOR1 signaling axis in patients with lung cancer.

## MATERIALS AND METHODS

### Mouse samples collection

Lung hyperplasia (Hyp), adenoma (Ad), adenocarcinoma (AdCa) and non-neoplastic lung (4-OHT+N) tissues (at least 10^4^ cells per sample) were obtained by laser-assisted microdissection (LMD; Leica Microsystems, Milan, Italy) from formalin-fixed and paraffin embedded (FFPE) lungs of K-Ras^(+/LSLG12Vgeo);RERTn(ert/ert)^ mice at 2 (*n* = 2), 5 (*n* = 2) and 9 (*n* = 4) months following K-Ras^v12^ oncogene induction by 4-OHT intraperitoneal injection (0.5 mg per dose, 3 doses per week, for 2 weeks). Hyperplastic lung lesions were identified in animals after 2, 5 and 9 months from oncogene expression. Adenomas were present in mice after 5 and 9 months following 4-OHT administration. Adenocarcinomas were detected in animals after 9 months from oncogene induction. Aged-matched normal lung tissues (N) were also purified from aged-matched littermates at 2, 5 and 9 months of age without K-Ras^v12^ oncogene induction (Supplementary Table 10).

Lung tissue morphology was evaluated on haematoxylin and eosin (H&E) stained sections to confirm presence of lesions or of normal lung tissue. All experiments involving mice were approved by an Institutional Animal Care and Use Committee.

### Lung cancer patients

A series of 113 non-small cell lung cancer (NSCLC) patients who underwent surgery for therapeutic purposes at Fondazione IRCCS Ca’ Granda-Ospedale Maggiore Policlinico Hospital (Milan, Italy) were enrolled for this study. The research was approved by the Fondazione IRCCS Ca’ Granda-Ospedale Maggiore Policlinico Ethical committee. All clinical investigation has been conducted according to the principles expressed in the Declaration of Helsinki and data were analyzed anonymously. For all patients clinico-pathological correlates were available. Disease-free survival status was available for 57 patients, whereas overall survival records were available for the entire cohort. Patients’ characteristics are detailed in Supplementary Table 11. Lung cancer tissues were available from all patients whereas non-neoplastic counterparts at a minimum distance from tumor of 5 cm were available for 57 patients. For all lung cancer samples, a pathologist (SB or SF) confirmed on histological sections that epithelial tumor cells represented at least 80% of the tissue.

Tumor bulk and periphery (invasive front) were isolated by laser-assisted microdissection from 16 cases of NSCLC.

### Cell cultures

The human lung cancer cell line A549 was purchased from American Type Culture Collection (ATCC, Teddington, UK). Cells were cultured in RPMI supplemented with 10% FBS and 1 % of Penicillin and Streptomycin (all from Thermo Fisher Scientific, Waltham, MA, USA) and maintained at 37°C and 5% CO_2_. For miRNA transfection experiments, A549 cells were seeded at 2×10^6^ cells/ml in 6-wells plates and transfected with miR-494-3p Mimic (100 nM, Sigma Aldrich, Milan, Italy), miR-494-3p Inhibitor (150 nM, Sigma Aldrich) or with a Mimic/Inhibitor control (50 nM, Sigma Aldrich) in the presence of 5 µl of Lipofectamine 2000 (Thermo Fisher Scientific) in 1ml of OptiMem Medium (Thermo Fisher Scientific). After transfection, cells were maintained in complete medium for 48 to 72 h, and processed for individual experiments.

### Side population analysis

Side population (SP) analyses were performed as already described [17, 40]. Briefly, transfected A549 cells were resuspended at 1×10^6^ cells/ml in pre-warmed DMEM supplemented with 2% FBS and 10mM HEPES (all from Thermo Fisher Scientific). Hoechst 33342 dye was added at the final concentration of 5 µg/ml in presence or absence of Verapamil (50 µM, Sigma Aldrich) and cells were incubated at 37°C for 2h with intermittent shaking. At the end of the incubation, cells were washed by centrifugation at 4°C with ice-cold HBSS (Thermo Fisher Scientific) and resuspended at the final concentration of 2×10^7^ cells/ml with cold HBSS w/o Ca^2+^/Mg^2+^ supplemented with 2% FBS and 10mM HEPES. In order to exclude dead cells, propidium iodide (PI, Sigma Aldrich) was added at the final concentration of 5µg/ml. SP analyses and cell sorting were conducted on FACSAria II SORP (Becton Dickinson, San Josè, CA, USA) equipped with a FACSDiva software (version 7.0). The first FSC-H versus FSC-A dot plot was used to exclude doublet cells from the analysis. The gate on SFC-A and SSC-A in the second dot plot was used to exclude debris. The third dot plot reporting SSC-A and PI fluorescence allowed the exclusion of dead cells from the analysis of SP without affecting the Hoechst 33342 profile. PI florescence was excited with a blue laser emitting at 488 nm and collected at 620-640 nm. The final dot plot with the Hoechst blue and red fluorescence shown on a linear scale was used to identify the SP (Supplementary Figure 5). The Hoechst 33342 dye was excited with ultra violet laser emitting at 355 nm and its emission fluorescence was dual-wavelength analyzed (Hoechst blue, 425–475 nm; Hoechst red, 650–700 nm). The SP was the final population that disappeared in the samples treated with Verapamil (Supplementary Figure 5A, B) as described [17, 40].

### Transcription factor array

A549 cells were reverse-transfected with Mimic/Inhibitor control and miR-494-3p Mimic/Inhibitor (26 nM) on a 96-wells plate pre-coated with reporter genes for 10 transcription factors (TF), as well as positive and negative controls (Cignal Finder Reporter array, Signal Transduction 10 Pathways, SaBiosciences Corp., Frederick, MD; Supplementary Table 9) as described [17]. Cells were harvested after 72 h and dual luciferase emission was assessed using a Dual-Glo Luciferase Assay (Promega Corporation, Madison, WI, USA) and a luminometer. Firefly luciferase emission was normalized on Renilla for each sample. Values of negative controls were used to set the threshold for TF activity. Relative pathway activation was calculated in miR-494-3p over expressing cells compared to control and a *p*-value < 0.05 were set as cut-off for significant difference.

### Migration assay

After 48h from miRNA Mimics transfection, a wound was created in the monolayer of A549 cells using a P200 micropipette tip. Cells were washed with PBS and incubated in complete medium for 24h at 37°C and 5%CO_2_. To measure the wound closure, three random pictures were taken at 50x magnification when the scratch was performed (T0) and after 24h (T24). The migration distance was determined as reduction in the wound gap using NIH Image-J software, as already described [2].

### Spheres formation

After 48h of transfection with miR-494-3p Mimic and control, A549 cells (5×10^3^ cells/ml) were seeded in poly-HEMA (20mg/ml, Sigma Aldrich) coated 6-wells plates in serum-free DMEM medium supplemented with 20 ng/ml human recombinant basic fibroblast growth factor (bFGF) and 20 ng/ml epidermal growth factor (EGF; all from Thermo Fisher Scientific). The medium was supplemented with fresh growth factors every 2 days until the cells started to grow and form floating tumor spheres. Tumor spheres were analyzed after 7 days.

### *In vivo* tumor growth and metastatic dissemination analyses

Fourteen male athymic nude-Foxn1^nu^ mice (Harlan Laboratories Srl, Udine, Italy) at 6 weeks of age were injected subcutaneously into the lower flank with 5×10^5^ A549 cells transfected with a Mimic control (Ctrl) or a miR-494-3p Mimic in a total volume of 200 μl of sterile PBS (seven mice per condition). Tumor growth was monitored by caliper and animals were euthanized after 18 days. Tumor volume (cm^3^) was calculated using the formula: Volume = (width)^2^ × length/2. Xenografts were formalin-fixed and paraffin embedded for histological examination.

Twenty-six male athymic nude-Foxn1^nu^ mice (Harlan Laboratories Srl) were injected intravenously into the lateral tail vein with 5×10^5^ A549 cells transfected with a Mimic control (Ctrl)or a miR-494-3p Mimic in a total volume of 200 μl of sterile PBS (13 mice per condition). The onset of metastatic tumor foci was monitored using magnetic resonance image (MRI) at day 10 and day 17. At mice sacrifice (day 18), lungs and liver were collected and processed following standard histopathological procedures (FFPE). Metastatic foci to the lung were then analyzed using Leica IM 500 V5 software (Leica Microsystems) and their number and area were scored and recorded by AF, VV and SB.

All experiments involving animals were approved by an Institutional Animal Care and Use Committee (IACUC) at Nerviano Medical Sciences, in compliance with the Italian Ministry of Health.

### MRI *in vivo* acquisition

MRI scans were acquired on anesthetized animals (isofluoran gas anaesthesia) using a Bruker Pharmascan instrument operating at 7.0 T magnetic field. Mice were positioned prone on the animal bed and inserted in the radiofrequency coil (38 mm i.d.) inside the magnet. After scout transverse imaging for correct positioning, spin echo (MSME) coronal images were acquired to cover the whole lungs. Acquisition parameters were as follows: TR/TE = 600/12 ms; 4 averages, FOV = 4*4, slice thickness: 0.55 mm, 22 slices. The acquisition was triggered to the animal respiratory cycle, in order to reduce thoracic movement artifacts. The whole in vivo examination lasted around 30 minutes per animal.

All experiments involving animals were approved by an Institutional Animal Care and Use Committee (IACUC) at Nerviano Medical Sciences, in compliance with the Italian Ministry of Health.

### Genomic DNA extraction and bisulfite treatment

Genomic DNA was purified from patients’ frozen samples using DNeasy Blood and Tissue Kit following the manufacturer's instructions (Qiagen, Hilden, Germany). DNA was quantified by Qubit 2.0 fluorimeter (Thermo Fisher Scientific) and bisulfite-modified using Epitect Bisulfite Kit (Qiagen), according with the manufacturer's protocol. Bisulfite converted DNA was eluted in 20 μl and used for HRM analysis.

### Differential high resolution melting analysis

PCR amplification and HRM analysis were conducted using a Rotor-Gene 6000 (Corbett Research, Sydney, Australia) instrument. Primers were designed by Methyl Primer Express software (Thermo Fisher Scientific) in order to amplify a 340 bp product containing 16 CpG sites at the intergenic differentially methylated region (IG-DMR) of human DLK1-DIO3 locus. The primer sequences were as follows: IG-Met4F: 5’-GGGAATTGGGGTATTGTTTATA-3’ and IG-Met3R: 5’-TAACCAATTACAATACCACAAAATTAC-3’. PCR amplification was carried out in a final volume of 25 μl containing: 1X FastStart SYBR Green Master (Roche, Indianapolis, IN, USA), 360 nM of each primer and 2 ng of bisulfite treated DNA template. The thermal profile was 10 min at 95° C followed by 40 cycles including 30 sec at 95° C, 30 sec at 59°C, 30 sec at 72° C. HRM was performed ramping from 69° C to 85° C and rising by 0.1° C every 2 sec.

The normalization of melting curves was performed for two normalization regions before and after the major fluorescence decrease using Rotor-Gene 6000 Series Software 1.7. The algorithm applied by the software permits the direct comparison of samples with different starting fluorescence levels. A differential profile was then evaluated for each sample by comparing the value of fluorescence at the melting point against the value of fluorescence of an unmethylated DNA control (Epitect PCR Control DNA Set - Qiagen) as described [41]. Quantification was calculated by interpolation on an external standard curve generated by serial dilutions of a control methylated DNA (100, 90, 75, 60, 50, 40, 25 and 10 % - Epitect PCR Control DNA Set - Qiagen). DNA from 12 matched lung normal and cancer tissues and a normal blood samples were investigated. All samples were analyzed in triplicate.

### Array-based comparative genome hybridization (a-CGH)

Genomic DNA was purified from 20 lung cancer samples and matched non-neoplastic counterparts using TRIzol Reagent (Thermo Fisher Scientific) following manufacturer's instructions. A-CGH analysis was performed using a 60-mer oligonucleotide probes technology (SurePrint G3 Human CGH 8×60K, Agilent Technologies, Santa Clara, CA, USA) according to supplier's information and as previously described [42]. Raw data were generated using Agilent Feature extraction and analysed by Cytogenomics 2.0.6.0. (Agilent Technologies). Copy number variations analysis was performed using ADAM2 algorithm. To improve the accuracy of the results the Diploid Peak Centralization algorithm was applied.

The aberration filter was set to detect a minimum number of 3 consecutive probes/region and the minimum absolute average Log Ratio (MAALR) was ± 0,25. A second analysis was run with a MAALR of ±0,15 and with a minimum number of 5 probes/region to detect low level mosaicism.

Copy number variations described in the Database of Genomic Variants (http://projects.tcag.ca/variation/) are not reported.

### Statistical analysis

For miRNAs profiling, log2-transformed data from miRNAs arrays were imported in BRB-ArrayTools software (https://brb.nci.nih.gov/BRB-ArrayTools/), quantile normalized and filtered to exclude miRNAs with low variability within samples pools (less than 20% of expression data with at least a 1.5 -fold change in either direction from gene's median value). Four hundred and eighty-six miRNAs were available for further analyses. In order to identify miRNAs potentially deregulated during cancer progression, we performed a Class Comparison analysis between normal (N), non-neoplastic (4-OHT+N), hyperplastic (Hyp), adenomatous (ad) and adenocarcinoma (Adca) lesions. Results were considered significant if the p-value was less than 0.05 and the fold-change was at least 2. For scatterplots generation, a fold-change of 10 was chose as cut-off for miRNA differential expression.

For both overall and disease-free survival analyses, the Kaplan-Meier method was used. NSCLC patients were categorized into “high” or “low” expressor group if their miRNA level was above or below the miRNA median value, or if they upregulated more than 8 chromosome 14q32 miRNAs (>8) or not (≤ 8 miRNAs).

Differences in miRNAs expression levels between matched lung tumor and normal samples were analyzed by paired Student's *t* test (Prism 4.0, GraphPad Inc, La Jolla, CA, USA).

Receiver operating characteristic (ROC) curve analysis was used to identify the best ABCG2 value able to discriminate between tumors with elevated expression of at least nine 14q32 miRNAs from cases with low 14q32 miRNAs abundance using the Youden associated criterion (J index = 0.578, MedCalc Software, Ostend, Belgium) as described [43].

Correlation between miR-494-3p and pri-miR-494 levels has been analyzed using the Spearman Rank Correlation. Differences in DNA methylation between tumor and normal tissues were analyzed by Mann-Whitney U test. Correlation between pri-miR-494 and submicroscopic genomic alteration of DLK1-DIO3 region was analyzed using Fisher's exact test. Differences in gene expression among A549 cells transfected with a miR-494-3p Mimic or a control were analyzed by Mann-Whitney U test. Correlation between miR-494-3p and pri-miR-494 levels has been analyzed using the Spearman Rank Correlation. Correlation between pri-miR-494 and submicroscopic genomic alteration of DLK1-DIO3 region was analyzed using Fisher's exact test. All analyses were performed using Prism 4.0 software (GraphPad Inc)

Ingenuity Pathway system (Ingenuity Systems Inc., Redwood City, CA, USA) was used to infer on cellular networks with biological significance using as dataset the analyzed genes (Supplementary Table 8) in A549 cultures overexpressing the miR-494-3p or a control construct.

For *in vitro* and *in vivo* experiments, differences among samples with miR-494-3p overexpression or control were analyzed using the unpaired Student's *t* test or the Mann-Whitney U test as indicated (Prism 4.0, GraphPad Inc).

RNA purification and retrotranscription, miRNAs and genes expression analyses, immunoblotting and immunohistochemical procedures and Boyden Chamber Assay are detailed in the Supplementary Methods.

## SUPPLEMENTARY MATERIALS FIGURES AND TABLES





## Abbreviations

4-OHT: 4-Hydroxytamoxifen
4-OHT+N: non-neoplastic lung tissue after K-Ras activation
Ad: Adenoma
AdCa: Adenocarcinoma
a-CGH: array-based Comparative Genomic Hybridization
Chr: Chromosome
Ct: Cycle threshold
DAB: 3,3’-Diaminobenzidine
EMT: Epithelial-Mesenchymal Transition
FFPE: Formalin-Fixed and Paraffin-Embedded
Hyp: Hyperplesia
H&E: Haematoxylin and Eosin
HRM: High Resolution Melt
IG-DMR: Intergenic differentially methylated region
MRI: Magnetic Resonance Image
N: normal lung tissue (without K-Ras^v12^ oncogene induction by 4-OHT administration)
NICD: Notch Intracellular Domain
NSCLC: Non Small Cell Carcinoma
NSP: Non-SP
PVDF: Polyvinylidene Fluoride
ROC: Receiving Operating Characteristic
RQ: Relative Quantity
RIPA Buffer: Radioimmunoprecipitation Assay Buffer
RT: Reverse Transcription
SP: Side Population
T0: time 0
T24: after 24h
TF: Transcription Factors
