# Supplementary material for: miR-494-3p is a novel tumor driver of lung carcinogenesis
Source: Oncotarget. 2016 Dec 14;8(5):7231–47. doi: 10.18632/oncotarget.13933 (PMC5352317; doi:10.18632/oncotarget.13933)
Supplement: Supplementary file 1 [file oncotarget-08-7231-s001.pdf]

miR-494-3p is a novel tumor driver of lung carcinogenesis

Supplementary Material

Supplementary Information includes Supplementary Methods, Figures (1-5), Tables (1-12) and References.

Supplementary Methods

RNA purification. For laser-assisted microdissected samples, total RNA was purified using the MasterPure RNA Purification Kit (Epicentre Biotechnologies, Madison, WI, USA) as described [1]. For the K-Ras$^{(+/LSLG12Vgeo);RERTn(ert/ert)}$ mouse model, RNA was pooled according to age (2, 5 or 9-months) and group (4-OHT induced or not, i.e. controls, animals). Therefore the following pools (n=12) were created: non-neoplastic lung tissues after 4-OHT induction (4-OHT+ N) at 2, 5 and 9 months; hyperplastic lesions (Hyp) at 2, 5 and 9 months; adenoma lesions at 5 and 9 months; adenocarcinoma at 9 months (AdCa); normal lung tissues from littermates without 4-OHT administration at 2, 5 and 9 months.. For frozen human normal and tumor samples, total RNA was isolated from about 50 mg of tissue using TRIZOL reagent (Thermo Fisher Scientific, Waltham, MA, USA) according to the supplier's protocol. For all samples, total RNA was quantified spectrophotometrically.

miRNA Reverse Transcription and detection. For mouse miRNA profiling, 50ng of total RNA per sample pool were reverse transcribed using the TaqMan MicroRNA Reverse Transcription Kit with the Megaplex RT Primers Rodent Pool A and B v.3.0 and then preamplified using the TaqMan PreAmp Master Mix with the Megaplex PreAmp Primers Rodent Pool A and B v.3.0, according to the manufacturer' specifications (Thermo Fisher Scientific) miRNAs low density array was performed using TaqMan Array miRNA Cards A and B set v3.0 (Thermo Fisher Scientific) which allowed specific quantification of 641 mature miRNAs as described [2]. miRNA relative quantities (RQ) were obtained using the geometrical mean of three reference small RNAs (U6, snoRNA135 and snoRNA202) as normalization factor. RQs were then median-normalized and log2 transformed for statistical analyses.

For individual miRNAs analyses, 100 ng of total RNA was reverse transcribed using the TaqMan MicroRNA Reverse Transcription Kit (Thermo Fisher Scientific). Expression levels of miRNAs were analyzed in duplicate using gene-specific primers and TaqMan

probes (Supplementary Table 12) and the ABI Prism 7900HT sequence detection system (Thermo Fisher Scientific). Targets raw data (Ct values) were converted into relative quantities using the $2^{\wedge\text{-DCt}}$ formula and then median-normalized and log2-transformed for statistical analysis. MammaryU6 and RNU48 were used as reference transcripts for relative quantification of miRNAs as described [1].

RNA Reverse Transcription and Gene expression analyses. For gene expression analyses, 1 ug of total RNA was retrotranscribed using the High-Capacity cDNA Reverse Transcription Kit (Thermo Fisher Scientific) after DNA digestion by Deoxyribonuclease I, Amplification Grade (Thermo Fisher Scientific). Expression levels of selected genes (Supplementary Table 8) and pri-miR-494 (Hs04225959_pri) were analyzed in duplicate using gene-specific primers and TaqMan (Thermo Fisher Scientific). Targets raw data (Ct values) were converted into relative quantities using $2^{\wedge\text{-DCt}}$ formula and then median-normalized and log2-transformed for statistical analysis. β-2 Microglobulin was used as reference gene for targets' relative quantification (RQ).

Immunoblotting. For immunoblotting, aliquots of A549 cells were harvested at 72h post-transfection and solubilized in 150µl of RIPA buffer supplemented with 1x complete protease inhibitor cocktail (Roche, Indianapolis, IN, USA) and phosphatase inhibitor (Sigma Aldrich, Milan, Italy). Cell extracts (50 µg) were separated on 12% SDS-polyacrylamide gels by electrophoresis and transferred to PVDF membranes (Millipore, Billerica, MA, USA). Extracts were probed with 1 µg/µl of antibodies against β-Tubulin (Sigma-Aldrich), Notch1 (Santa Cruz Biotechnologies, Dallas, TX, USA), NICD (Cell Signaling Technologies, Danvers, MA, USA), CDKN1A (Sigma Aldrich), AKT1, AKT2, pAKT1 (Ser 473), S6RP and pS6RP (all from Cell Signaling Technologies), pAKT2 (Ser474) (Abcam, Cambridge, UK), PDK1 (Abcam) or pPDK1 T346 [3]. Reactive bands were visualized with ECL Plus reagents (GE Healthcare, Milan, Italy).

Immunohistochemical analysis. Immunostaining was performed for pS6RP (1:1000; Cell Signaling Technologies). Endogenous mouse IgG and non-specific background were blocked using Rodent Block M (Biocare Medical, Concord, CA, USA). Binding of the individual primary antibody was detected with a secondary antibody of appropriate specificity, and visualized by DAB followed by counterstaining with hematoxylin using the UltraView DAB Detection Kit and Benchmark Ultra instrument (Ventana Medical Systems, Inc). Negative controls were prepared in the absence of primary antibody and included in each reaction. Two investigators (AF and VV) independently examined and scored all slides. When discrepancies occurred, the cases were reviewed jointly until a consensus was reached. A two-score system for percentage of positive cells and intensity of staining was used to quantify the reactivity for pS6RP. The intensity of staining was expressed in a scale of 0 (absent staining) to 3 (strong staining) [4]. Representative images were obtained using an DMD108 system (Leica Microsystems).

Boyden Chamber Assay. Boyden chamber was used to assess A549 cells migration after 48h from miRNA Mimics transfection. A549 cells (10^4) were seeded in the upper compartment of the Boyden chamber (Sigma Aldrich) in serum free-RPMI medium. RPMI medium supplemented with 10% FBS and 1 % of Penicillin and Streptomycin (all from Thermo Fisher Scientific) was added to the lower compartment of the Boyden chamber. Cells were allowed to migrate through the 8µm membrane pores for 24h at 37°C and 5%CO_2. Membrane was fixed with 100% Methanol (Sigma Aldrich) and stained with 1% Toluidine Blue (Sigma Aldrich). The number of migrated cells was determined by direct cell count at the microscope.

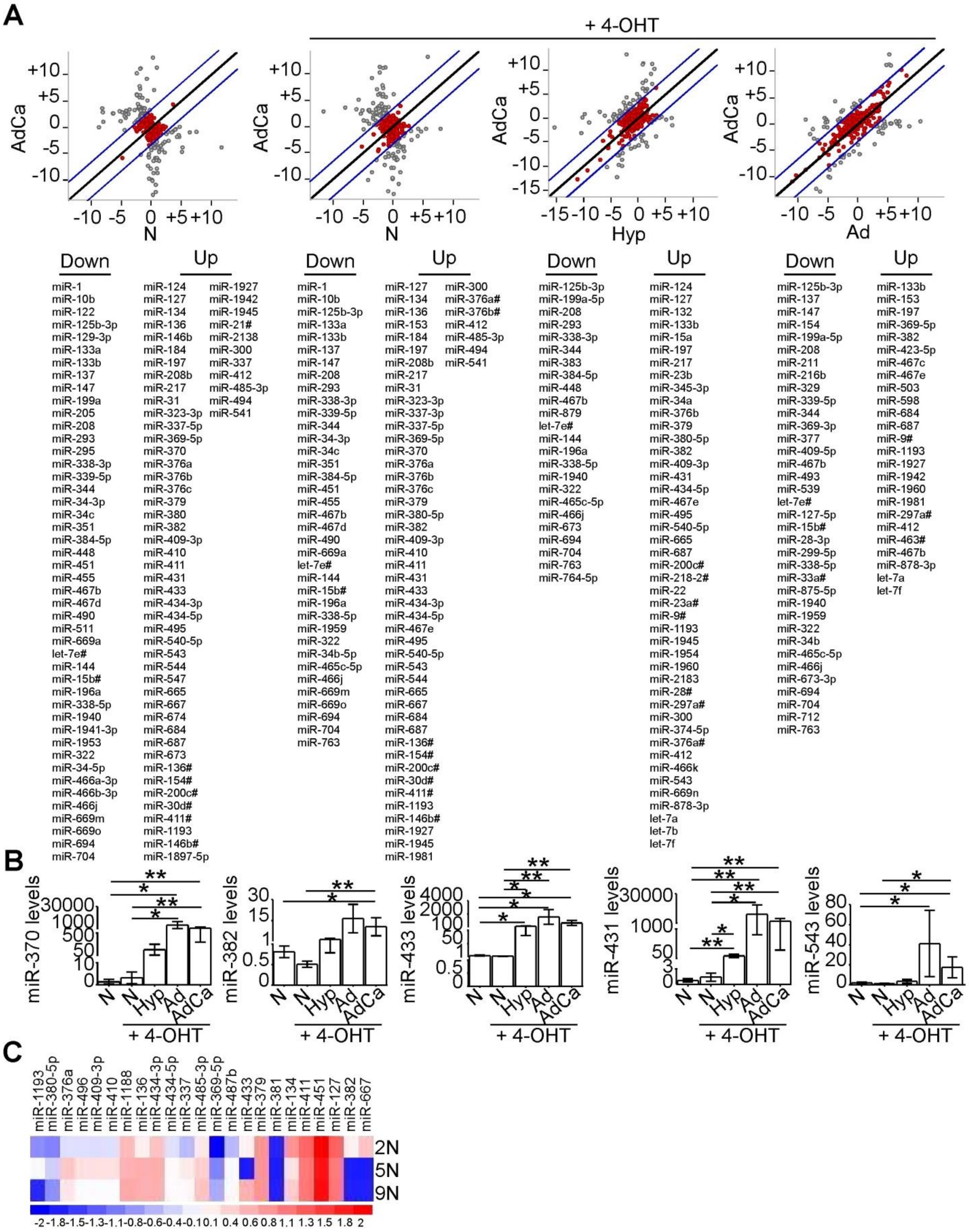

Supplementary Fig. 1 miRNAs expression profile in K-Ras$^{(+/LSLG12Vgeo);RERTn(ert/ert)}$ mice tumorigenesis. **A**) Scatterplot analyses between AdCa (murine lung adenocarcinoma) and non-neoplastic lung tissues (N) with (+4-OHT) or without Tamoxyfen administration, hyperplasia (Hyp) and adenoma (Ad). Up and downregulated miRNAs are listed below the charts. **B**) Chromosome 12qF1 miRNAs expression validation by single qPCRs. **C**) Chromosome 12qF1 miRNAs analysis in non-neoplastic lung tissues of mice at different ages (2, 5 and 9 months) without Tamoxyfen administration.

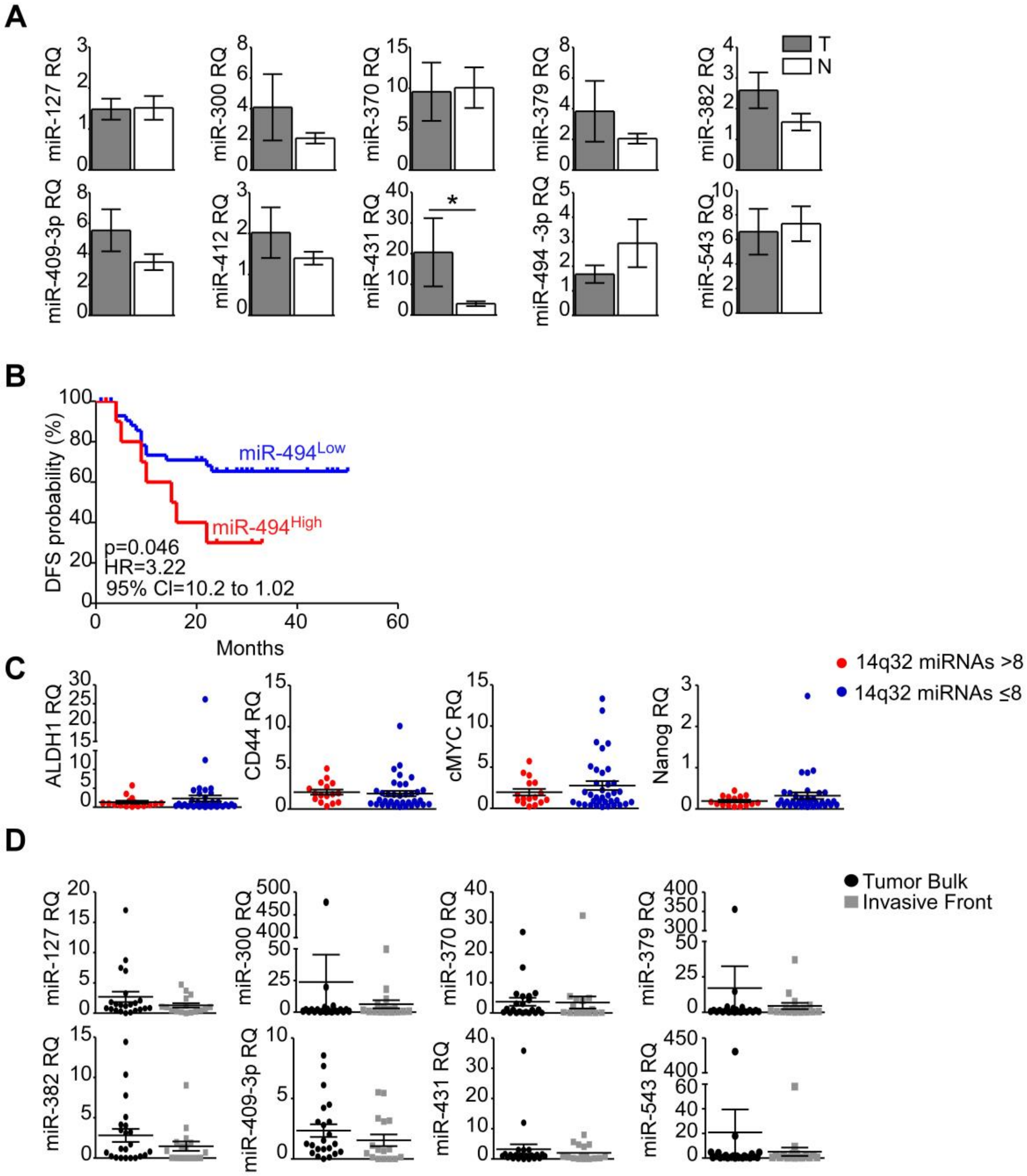

Supplementary Fig. 2. Chromosome 14q32 miRNAs analysis in human samples. **A**) Expression analysis of 10 miRNAs belonging to 14q32 locus in tumor (T) and matched normal (N) tissues derived from patients with NSCLC. *, p=0.01 by paired *t* test. **B**) Kaplan-Meier curve of NSCLC patients' overall survival according to high or low expression of miR-494-3p (p=0.04; HR, Hazard Ratio; CI, Confidential Interval). **C**) ALDH1, CD44, cMyc and Nanog gene expression was analyzed in NSCLC tissues of patients with (>8) or without (≤8) major upregulation of 14q32 miRNAs. **D**) A subset of Chr. 14q32 miRNAs (n=10) was investigated in tumor samples corresponding to the invasive front or central area. miR-412 was not expressed.

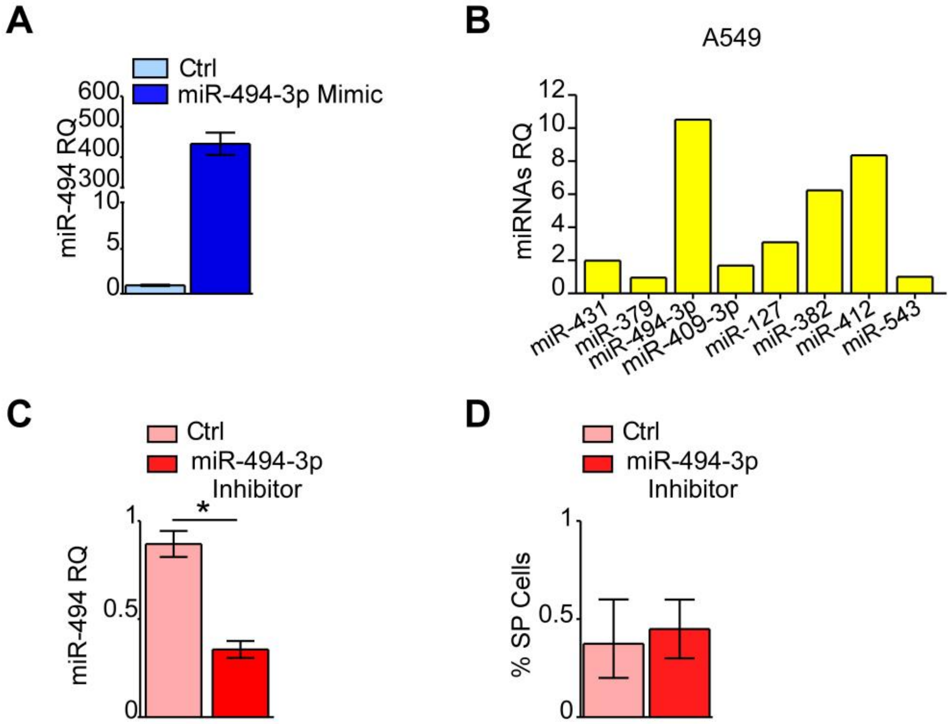

Supplementary Fig.3. A) miR-494-3p expression levels 10 days after A549 cells transfection with a miR-494-3p Mimic or a control construct (Ctrl). **B**) Chromosome 14q32 miRNAs analysis (n=10) in A549 lung cancer cells. miR-300 and miR-370 were not expressed. **C**) miR-494-3p expression levels 72h after A549 cells transfection with a miR-494-3p Inhibitor or a control construct (Ctrl). *, p= 0.03 by unpaired *t* test. **D**) SP cells analysis in A549 with reduced miR-494-3p levels, compared to control.

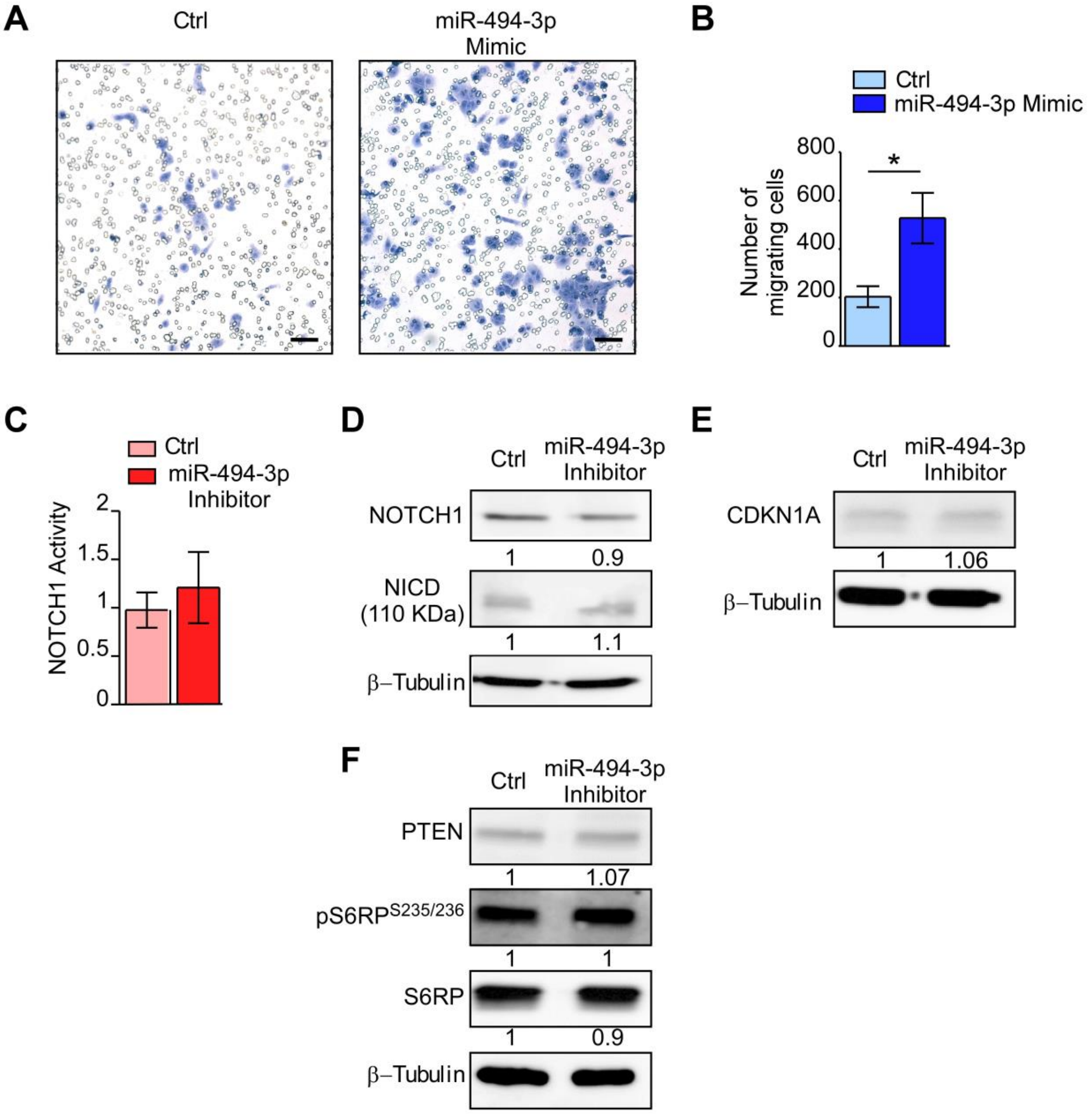

Supplementary Fig.4. A, B) A549 cells transfected as indicated were allowed to migrate for 24h using a Boyden chamber assay. Representative images of migrated A549 cells are shown (**A**; bars, 100 µm) and quantified in **B**. *, p=0.02 by unpaired Student's *t* test. **C**) Quantification of NOTCH1 activity in A549 transfected with a miR-494-3p or a control Inhibitor. **D**) Western blot of NOTCH1 and NICD in A549 cells transfected with a miR-494-3p or a control Inhibitor. **E, F**) Indicated proteins contents were analyzed by western blotting in A549 cells transfected with a miR-494-3p Inhibitor or a control construct.

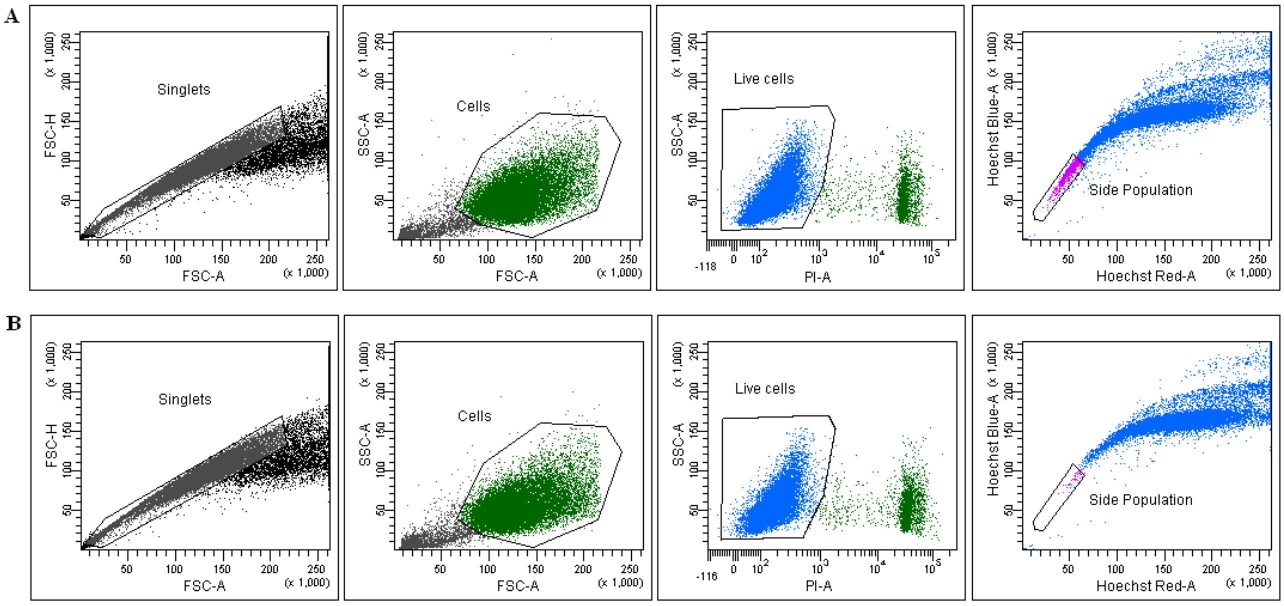

Supplementary Fig.5. FACS gating strategy and Side Population (SP) Analysis. The upper panels (**A**) represent A549 cells stained with Hoechst 33342 whereas the lower panels (**B**) show A549 cells incubated with Hoechst 33342 and Verapamil. The plots show (from left-to-right): gating strategy for doublets exclusion, debris exclusion, live cells identification (by PI staining) and SP analysis (by Hoechst 33342 staining), respectively.

Supplementary References

1. Vaira V, Faversani A, Dohi T, Montorsi M, Augello C, Gatti S, Coggi G, Altieri DC, Bosari S. miR-296 regulation of a cell polarity-cell plasticity module controls tumor progression. Oncogene. 2012; 31(1): 27-38.
2. Forno I, Ferrero S, Russo MV, Gazzano G, Giangiobbe S, Montanari E, Del Nero A, Rocco B, Albo G, Languino LR, Altieri DC, Vaira V, Bosari S. Deregulation of MiR-34b/Sox2 Predicts Prostate Cancer Progression. PLoS One. 2015; 10(6): e0130060.
3. Chae YC, Vaira V, Caino MC, Tang HY, Kossenkov AV, Ottobrini L, Martelli C, Lucignani G, Bertolini I, Locatelli M, Bryant KG, Ghosh JC, Lisanti S, Ku B, Bosari S, Languino LR, Speicher DW, and Altieri DC. Mitochondrial Akt regulation of hypoxic metabolic reprogramming. Cancer Cell. 2016; 30(2): 257-72
4. Russo MV, Faversani A, Gatti S, Ricca D, Del Gobbo A, Ferrero S, Palleschi A, Vaira V, Bosari S. A new mouse avatar model of non-small cell lung cancer. Frontiers in Oncology. 2015; 5: 52.
