# Supplementary material for: miR-494-3p is a novel tumor driver of lung carcinogenesis
Source: Oncotarget. 2016 Dec 14;8(5):7231–47. doi: 10.18632/oncotarget.13933 (PMC5352317; doi:10.18632/oncotarget.13933)
Supplement: Supplementary file 2 [file oncotarget-08-7231-s002.docx]

Supplementary Table 1: miRNAs expression analysis in non-neoplastic lung tissues derived from K-Ras^(+/LSLG12Vgeo);RERTn(ert/ert)^ mice. N, normal lung tissue; 4-OHT+N, non-neoplastic lung tissue after Tamoxifen administration.

| **UniqueID** | **Geom mean of intensities in class 1 4-OHT+N** | **Geom mean of intensities in class 2 N** | **Fold-change (4-OHT+N /N)** | **Parametric p-value** |
| --- | --- | --- | --- | --- |
| mmu-miR-1941-3p-121130_mat | 0.59 | 1.61 | 0.37 | 0.0009664 |
| mmu-miR-489-4378114 | 0.6 | 1.34 | 0.45 | 0.0015928 |
| mmu-miR-199a-5p-4373272 | 0.027 | 3311.57 | 8.20E-06 | 0.0017427 |
| mmu-miR-682-4381081 | 1.12 | 0.32 | 3.52 | 0.0170997 |
| mmu-let-7e-4395517 | 0.66 | 1.44 | 0.45 | 0.0261079 |
| mmu-miR-193-4395361 | 0.91 | 2.03 | 0.45 | 0.0377009 |
| mmu-miR-132-4373143 | 0.29 | 1.05 | 0.28 | 0.0382354 |
| mmu-miR-328-4373049 | 0.75 | 1.66 | 0.45 | 0.0411453 |
| mmu-miR-1-4395333 | 1.74 | 4.88 | 0.36 | 0.0437294 |
| mmu-miR-449a-4373207 | 2.89 | 1.01 | 2.85 | 0.0481264 |

Supplementary Table 2: miRNAs expression analysis in normal lung and hyperplastic tissues derived from K-Ras^(+/LSLG12Vgeo);RERTn(ert/ert)^ mice. N, normal lung tissue; Hyp, hyperplasia.

| **UniqueID** | **Geom mean of intensities in class 1 Hyp** | **Geom mean of intensities in class 2 N** | **Fold-change (Hyp/N)** | **Parametric p-value** |
| --- | --- | --- | --- | --- |
| mmu-miR-145-4395389 | 0.22 | 1.77 | 0.12 | 1.00E-07 |
| mmu-miR-143-4395360 | 0.37 | 1.49 | 0.25 | 5.00E-07 |
| mmu-miR-142-5p-4395359 | 0.5 | 1.01 | 0.49 | 6.20E-06 |
| mmu-let-7a-4373169 | 2.60E-05 | 1.11 | 2.30E-05 | 0.0002143 |
| mmu-miR-133b-4395358 | 0.00072 | 3.86 | 0.00019 | 0.0005017 |
| mmu-miR-466b-3-3p-002500 | 0.9 | 182 | 0.005 | 0.0005157 |
| mmu-miR-339-5p-4395368 | 0.00045 | 1.19 | 0.00038 | 0.000699 |
| mmu-miR-146b-4373178 | 2.27 | 0.14 | 15.79 | 0.0011028 |
| mmu-miR-21#-002493 | 1.31 | 0.26 | 5.14 | 0.0011055 |
| mmu-miR-351-4373345 | 0.0017 | 1.73 | 0.00096 | 0.0013034 |
| mmu-miR-146b#-002453 | 0.93 | 0.14 | 6.47 | 0.0013764 |
| mmu-miR-485-3p-001943 | 1.42 | 0.22 | 6.5 | 0.001444 |
| mmu-miR-411-4381013 | 1.82 | 0.3 | 6.17 | 0.0015465 |
| mmu-miR-184-4373113 | 3.39 | 0.054 | 63.19 | 0.0017148 |
| hsa-miR-183#-002270 | 1.76 | 0.43 | 4.08 | 0.0017466 |
| mmu-miR-34b-3p-4395748 | 0.18 | 1.85 | 0.095 | 0.0019784 |
| mmu-miR-335-5p-4373045 | 0.33 | 1.69 | 0.19 | 0.0020869 |
| mmu-miR-142-3p-4373136 | 0.27 | 1.38 | 0.19 | 0.0021797 |
| mmu-miR-200c-4395411 | 1.52 | 0.3 | 5.08 | 0.0023581 |
| mmu-miR-434-3p-4395734 | 1.69 | 0.35 | 4.85 | 0.0026084 |
| mmu-miR-34b-001065 | 0.022 | 1.79 | 0.013 | 0.002792 |
| mmu-miR-455-4395585 | 0.0079 | 2.07 | 0.0038 | 0.0029202 |
| mmu-miR-10a-4373153 | 0.31 | 1.58 | 0.2 | 0.0032865 |
| mmu-miR-193#-002577 | 0.46 | 1.13 | 0.41 | 0.0032965 |
| mmu-miR-669a-4381091 | 0.23 | 38.24 | 0.006 | 0.0035888 |
| hsa-miR-136#-002100 | 1.2 | 0.31 | 3.83 | 0.0048208 |
| mmu-miR-1188-002866 | 1.48 | 0.47 | 3.13 | 0.0050657 |
| mmu-miR-669m-121190_mat | 0.49 | 19.46 | 0.025 | 0.0052042 |
| mmu-miR-133a-4395357 | 0.12 | 3.01 | 0.039 | 0.0058581 |
| mmu-miR-99a-4373008 | 0.32 | 1.07 | 0.3 | 0.0064862 |
| mmu-miR-126-5p-4373269 | 0.24 | 1.96 | 0.12 | 0.0065122 |
| mmu-miR-34c-4373036 | 0.022 | 1.86 | 0.012 | 0.0066208 |
| hsa-miR-93#-002139 | 1.12 | 0.55 | 2.04 | 0.007619 |
| hsa-miR-200c-000505 | 1.14 | 0.35 | 3.23 | 0.0077134 |
| mmu-miR-410-4378093 | 2.86 | 0.29 | 9.93 | 0.0078627 |
| hsa-miR-99b#-002196 | 1.67 | 0.5 | 3.3 | 0.0080215 |
| mmu-miR-155-4395701 | 1.05 | 0.34 | 3.06 | 0.0082305 |
| mmu-miR-720-001629 | 1.2 | 0.47 | 2.56 | 0.0083786 |
| mmu-miR-182-4395729 | 1.62 | 0.52 | 3.11 | 0.0086749 |
| mmu-miR-1982.2-121154_mat | 1.49 | 0.58 | 2.59 | 0.0087962 |
| mmu-miR-877#-002548 | 1.51 | 0.48 | 3.16 | 0.0092377 |
| mmu-miR-1971-121161_mat | 0.91 | 0.35 | 2.56 | 0.0093943 |
| mmu-miR-29b#-002497 | 1.67 | 0.4 | 4.16 | 0.0095152 |
| mmu-miR-100-4373160 | 0.38 | 1.24 | 0.31 | 0.0096915 |
| mmu-miR-467e-4395698 | 0.23 | 12.06 | 0.019 | 0.010021 |
| mmu-miR-674#-001956 | 1.44 | 0.35 | 4.09 | 0.0100236 |
| mmu-miR-541-002562 | 1.95 | 0.15 | 12.93 | 0.0100523 |
| mmu-miR-674-4395193 | 1.3 | 0.32 | 4.03 | 0.0116179 |
| mmu-miR-181c-4373115 | 0.33 | 0.83 | 0.4 | 0.0118556 |
| mmu-miR-193-4395361 | 0.5 | 2.03 | 0.24 | 0.012109 |
| mmu-miR-376a-4373347 | 2.29 | 0.23 | 9.89 | 0.0122227 |
| mmu-let-7i-4395332 | 0.42 | 1.08 | 0.39 | 0.0134759 |
| mmu-miR-296-5p-4373066 | 0.66 | 1.71 | 0.38 | 0.0134878 |
| mmu-miR-215-4373316 | 0.51 | 2.12 | 0.24 | 0.0136009 |
| mmu-miR-511-4395679 | 1.64 | 86.98 | 0.019 | 0.014203 |
| mmu-miR-301b-4395730 | 0.55 | 1.41 | 0.39 | 0.0146956 |
| mmu-miR-467a-4395717 | 0.36 | 0.93 | 0.39 | 0.0150291 |
| mmu-miR-494-001293 | 1.28 | 0.012 | 104.85 | 0.0151835 |
| mmu-miR-26a-4395166 | 0.25 | 1.63 | 0.15 | 0.0153708 |
| mmu-miR-384-5p-4395732 | 0.39 | 2.55 | 0.15 | 0.0158073 |
| hsa-miR-421-002700 | 1.47 | 0.57 | 2.58 | 0.0159036 |
| mmu-miR-125b-5p-4373148 | 0.35 | 2.25 | 0.16 | 0.01596 |
| mmu-miR-185-4395382 | 0.54 | 1.43 | 0.38 | 0.0160947 |
| mmu-miR-1-4395333 | 0.012 | 4.88 | 0.0024 | 0.0164094 |
| hsa-miR-324-3p-000579 | 1.53 | 0.57 | 2.67 | 0.0167455 |
| mmu-miR-1942-121136_mat | 1.57 | 0.24 | 6.55 | 0.0179546 |
| mmu-miR-1959-121132_mat | 1.28 | 0.19 | 6.55 | 0.0179546 |
| mmu-miR-300-000191 | 1.77 | 0.27 | 6.55 | 0.0179546 |
| mmu-miR-412-002575 | 1.77 | 0.27 | 6.55 | 0.0179546 |
| mmu-miR-1896-121128_mat | 1.96 | 0.31 | 6.42 | 0.0182475 |
| mmu-miR-1953-121159_mat | 0.24 | 1.89 | 0.13 | 0.0190392 |
| mmu-miR-10b-4395329 | 0.13 | 34.02 | 0.0038 | 0.0197833 |
| mmu-miR-450a-5p-4395414 | 0.26 | 2.14 | 0.12 | 0.0199809 |
| mmu-miR-328-4373049 | 0.5 | 1.66 | 0.3 | 0.0211921 |
| mmu-miR-1274a-121150_mat | 1.12 | 0.55 | 2.04 | 0.0212284 |
| mmu-miR-489-4378114 | 0.32 | 1.34 | 0.24 | 0.0231099 |
| mmu-miR-212-002551 | 1.15 | 0.57 | 2.03 | 0.0236049 |
| mmu-miR-431-4395173 | 7.58 | 0.19 | 39.01 | 0.0236274 |
| mmu-miR-361-4373035 | 0.37 | 1.18 | 0.31 | 0.0237951 |
| mmu-miR-26b-4395167 | 0.39 | 1.62 | 0.24 | 0.0243484 |
| mmu-miR-350-4395660 | 0.17 | 2.85 | 0.06 | 0.0248684 |
| mmu-miR-31-4373331 | 1.43 | 0.28 | 5.05 | 0.0255993 |
| mmu-miR-30c-4373060 | 0.39 | 1.6 | 0.25 | 0.025717 |
| hsa-miR-9#-002231 | 0.04 | 2 | 0.02 | 0.0300918 |
| mmu-miR-764-5p-002031 | 68.99 | 0.26 | 262.35 | 0.0309544 |
| hsa-miR-154#-000478 | 2.77 | 0.054 | 51.45 | 0.0328533 |
| mmu-miR-409-3p-4395443 | 3.07 | 0.2 | 15.71 | 0.0332526 |
| mmu-miR-323-3p-4395338 | 9.67 | 0.19 | 49.74 | 0.0336001 |
| mmu-miR-125a-5p-4395309 | 2.92 | 0.37 | 7.84 | 0.0342207 |
| mmu-miR-31#-002495 | 1.78 | 0.44 | 4.07 | 0.0353641 |
| mmu-miR-542-3p-4378101 | 0.22 | 1.15 | 0.19 | 0.0359876 |
| mmu-miR-29b-4373288 | 0.14 | 1.2 | 0.12 | 0.0386373 |
| mmu-miR-1941-3p-121130_mat | 0.25 | 1.61 | 0.16 | 0.0390614 |
| mmu-miR-1897-5p-121199_mat | 1.12 | 0.35 | 3.22 | 0.0422774 |
| mmu-miR-302a-4378070 | 10.79 | 0.38 | 28.03 | 0.0466657 |
| mmu-miR-140-4373374 | 0.44 | 1.15 | 0.39 | 0.0467748 |
| mmu-miR-137-4373301 | 0.15 | 1.54 | 0.096 | 0.0468686 |
| mmu-miR-544-4395680 | 2.01 | 0.08 | 24.99 | 0.0473627 |
| mmu-miR-101a-4395364 | 0.33 | 0.86 | 0.39 | 0.0475248 |
| mmu-miR-672-4395438 | 1.42 | 0.57 | 2.47 | 0.0491286 |
| mmu-miR-34c#-002584 | 0.18 | 1.78 | 0.1 | 0.049992 |

Supplementary Table 3: miRNAs expression analysis in normal lung tissue and adenoma derived from K-Ras^(+/LSLG12Vgeo);RERTn(ert/ert)^ mice. N, normal lung tissue; Ad, adenoma.

| **UniqueID** | **Geom mean of intensities in class 1 Ad** | **Geom mean of intensities in class 2 N** | **Fold-change (Ad/N)** | **Parametric p-value** |
| --- | --- | --- | --- | --- |
| mmu-miR-133a-4395357 | 0.045 | 3.01 | 0.015 | 2.90E-06 |
| mmu-miR-489-4378114 | 0.078 | 1.34 | 0.058 | 5.30E-06 |
| mmu-miR-335-5p-4373045 | 0.1 | 1.69 | 0.059 | 1.01E-05 |
| mmu-miR-126-5p-4373269 | 0.23 | 1.96 | 0.12 | 2.03E-05 |
| mmu-miR-142-5p-4395359 | 0.12 | 1.01 | 0.12 | 2.19E-05 |
| mmu-miR-145-4395389 | 0.21 | 1.77 | 0.12 | 3.05E-05 |
| mmu-miR-350-4395660 | 0.68 | 2.85 | 0.24 | 3.28E-05 |
| mmu-miR-199a-3p-4395415 | 0.37 | 1.56 | 0.24 | 6.37E-05 |
| mmu-miR-221-4373077 | 0.53 | 1.11 | 0.47 | 0.0002081 |
| mmu-miR-106b-4373155 | 0.43 | 0.91 | 0.47 | 0.0003468 |
| mmu-miR-301b-4395730 | 0.67 | 1.41 | 0.48 | 0.0003693 |
| mmu-miR-491-4381053 | 0.48 | 1.03 | 0.47 | 0.0003872 |
| mmu-miR-146a-4373132 | 0.36 | 0.75 | 0.47 | 0.0004885 |
| mmu-let-7c-4373167 | 0.67 | 1.4 | 0.48 | 0.0007603 |
| mmu-miR-351-4373345 | 5.00E-04 | 1.73 | 0.00029 | 0.0010104 |
| mmu-miR-669a-4381091 | 0.07 | 38.24 | 0.0018 | 0.0014767 |
| mmu-miR-146b#-002453 | 1.18 | 0.14 | 8.25 | 0.0016092 |
| mmu-miR-455-4395585 | 0.0024 | 2.07 | 0.0012 | 0.0016396 |
| hsa-miR-144-002676 | 8.00E-04 | 0.8 | 0.001 | 0.0017342 |
| mmu-miR-31-4373331 | 3.12 | 0.28 | 11.05 | 0.0018294 |
| mmu-miR-369-3p-4373032 | 17.98 | 0.27 | 67.77 | 0.0019975 |
| mmu-miR-31#-002495 | 2.91 | 0.44 | 6.64 | 0.002044 |
| hsa-miR-338-000548 | 0.012 | 0.92 | 0.013 | 0.0021001 |
| mmu-miR-451-4373360 | 0.082 | 2.21 | 0.037 | 0.0021094 |
| mmu-miR-878-3p-002541 | 0.0024 | 0.7 | 0.0033 | 0.0030915 |
| mmu-miR-384-5p-4395732 | 0.012 | 2.55 | 0.0045 | 0.0031617 |
| mmu-miR-466b-3-3p-002500 | 0.36 | 182 | 0.002 | 0.0034483 |
| mmu-miR-184-4373113 | 3.28 | 0.054 | 61.29 | 0.0036278 |
| mmu-miR-467e-4395698 | 0.07 | 12.06 | 0.0058 | 0.0036491 |
| mmu-miR-146b-4373178 | 1.73 | 0.14 | 12.01 | 0.0037036 |
| hsa-miR-9#-002231 | 0.025 | 2 | 0.012 | 0.0037914 |
| mmu-miR-34b-3p-4395748 | 0.098 | 1.85 | 0.053 | 0.0038241 |
| mmu-miR-143-4395360 | 0.25 | 1.49 | 0.17 | 0.0040471 |
| mmu-miR-199b-001131 | 0.19 | 0.93 | 0.21 | 0.004193 |
| mmu-miR-142-3p-4373136 | 0.23 | 1.38 | 0.17 | 0.0042033 |
| mmu-miR-669n-197143_mat | 1.48 | 0.45 | 3.32 | 0.0043319 |
| mmu-miR-200c-4395411 | 1.64 | 0.3 | 5.46 | 0.0049101 |
| hsa-miR-200c-000505 | 1.46 | 0.35 | 4.15 | 0.0051731 |
| mmu-miR-493-4395649 | 92.75 | 0.34 | 271.69 | 0.0060643 |
| mmu-miR-598-4395606 | 0.026 | 2.28 | 0.012 | 0.0061807 |
| mmu-miR-494-001293 | 8.29 | 0.012 | 676.91 | 0.0067841 |
| mmu-miR-485-3p-001943 | 3.64 | 0.22 | 16.61 | 0.0070956 |
| mmu-miR-365-4373194 | 0.17 | 3.1 | 0.053 | 0.0076186 |
| mmu-miR-293-4386754 | 0.022 | 0.75 | 0.029 | 0.0079116 |
| mmu-miR-511-4395679 | 0.5 | 86.98 | 0.0057 | 0.0080878 |
| mmu-miR-2135-241140_mat | 1.42 | 0.43 | 3.31 | 0.0083301 |
| mmu-miR-335-3p-4395296 | 0.12 | 0.75 | 0.16 | 0.008373 |
| mmu-miR-150-4373127 | 0.15 | 0.94 | 0.16 | 0.00909 |
| mmu-miR-193-4395361 | 0.37 | 2.03 | 0.18 | 0.010606 |
| mmu-miR-1-4395333 | 0.018 | 4.88 | 0.0036 | 0.0108808 |
| mmu-miR-448-4373206 | 0.037 | 2.5 | 0.015 | 0.0115172 |
| mmu-miR-674#-001956 | 1.87 | 0.35 | 5.29 | 0.01265 |
| mmu-miR-181a-4373117 | 0.31 | 0.96 | 0.33 | 0.0145604 |
| mmu-miR-669m-121190_mat | 0.2 | 19.46 | 0.01 | 0.0149752 |
| mmu-miR-1897-5p-121199_mat | 1.01 | 0.35 | 2.9 | 0.0150941 |
| mmu-miR-223-4395406 | 0.22 | 1.18 | 0.19 | 0.0152313 |
| mmu-miR-497-4381046 | 0.39 | 1.18 | 0.33 | 0.0159216 |
| mmu-miR-487b-001306 | 4.06 | 0.49 | 8.28 | 0.0160153 |
| mmu-miR-30c-4373060 | 0.53 | 1.6 | 0.33 | 0.0160299 |
| mmu-miR-10a-4373153 | 0.3 | 1.58 | 0.19 | 0.0162212 |
| mmu-miR-27a-4373287 | 0.4 | 1.18 | 0.34 | 0.01685 |
| mmu-miR-409-3p-4395443 | 30.17 | 0.2 | 154.19 | 0.0170718 |
| mmu-miR-26a-4395166 | 0.55 | 1.63 | 0.34 | 0.017241 |
| mmu-miR-26b-4395167 | 0.54 | 1.62 | 0.33 | 0.0173811 |
| mmu-miR-340-3p-4395370 | 0.54 | 1.6 | 0.33 | 0.0174515 |
| hsa-miR-340-000550 | 0.46 | 1.13 | 0.41 | 0.0175942 |
| hsa-miR-200b#-002274 | 2.76 | 0.26 | 10.5 | 0.0178199 |
| mmu-miR-136#-002512 | 3.76 | 0.35 | 10.71 | 0.0185614 |
| mmu-miR-182-4395729 | 1.39 | 0.52 | 2.67 | 0.0185635 |
| mmu-miR-30b-4373290 | 0.53 | 1.58 | 0.33 | 0.0187117 |
| mmu-miR-193#-002577 | 0.46 | 1.13 | 0.41 | 0.0187768 |
| mmu-miR-682-4381081 | 2.11 | 0.32 | 6.61 | 0.0189196 |
| mmu-miR-1954-121137_mat | 1.38 | 0.57 | 2.41 | 0.0191329 |
| mmu-miR-34c#-002584 | 0.072 | 1.78 | 0.041 | 0.0196634 |
| mmu-miR-34a-4395168 | 1.47 | 0.54 | 2.72 | 0.0203076 |
| mmu-miR-185-4395382 | 0.46 | 1.43 | 0.32 | 0.0210042 |
| mmu-miR-376a-4373347 | 7.95 | 0.23 | 34.37 | 0.0221179 |
| mmu-let-7a#-002478 | 0.59 | 1.41 | 0.42 | 0.0224844 |
| hsa-miR-189-000488 | 0.27 | 0.83 | 0.32 | 0.0232716 |
| mmu-miR-141-4373137 | 1.43 | 0.53 | 2.69 | 0.0235845 |
| mmu-miR-21#-002493 | 1.48 | 0.26 | 5.78 | 0.0236914 |
| mmu-miR-128a-4395327 | 0.47 | 2.26 | 0.21 | 0.0243076 |
| mmu-miR-125a-3p-4395310 | 1.43 | 0.53 | 2.67 | 0.024481 |
| mmu-miR-210-4373089 | 1.39 | 0.52 | 2.67 | 0.0250375 |
| mmu-miR-434-3p-4395734 | 7.43 | 0.35 | 21.38 | 0.02515 |
| mmu-miR-1941-5p-121140_mat | 0.058 | 1.59 | 0.036 | 0.0252596 |
| mmu-miR-450B-3P-002632 | 0.23 | 1.26 | 0.18 | 0.025926 |
| mmu-miR-1894-3p-241002_mat | 1.3 | 0.56 | 2.31 | 0.0260923 |
| mmu-miR-429-4373355 | 1.42 | 0.53 | 2.69 | 0.0261278 |
| mmu-miR-1968-121179_mat | 0.33 | 1.02 | 0.32 | 0.0263271 |
| mmu-miR-10b-4395329 | 0.039 | 34.02 | 0.0011 | 0.0274564 |
| mmu-miR-362-5p-002614 | 1.31 | 0.57 | 2.31 | 0.0289674 |
| mmu-miR-195-4373105 | 0.38 | 1.27 | 0.3 | 0.0298548 |
| mmu-miR-720-001629 | 0.97 | 0.47 | 2.07 | 0.032297 |
| mmu-miR-187-4373307 | 0.31 | 0.92 | 0.34 | 0.0325538 |
| mmu-miR-338-3p-4395363 | 0.035 | 1.9 | 0.019 | 0.0326912 |
| mmu-miR-2183-241095_mat | 1.07 | 0.52 | 2.06 | 0.0337657 |
| mmu-miR-449a-4373207 | 0.27 | 1.01 | 0.27 | 0.0338346 |
| hsa-miR-93#-002139 | 1.14 | 0.55 | 2.06 | 0.0339828 |
| mmu-let-7e-4395517 | 0.56 | 1.44 | 0.39 | 0.0344343 |
| hsa-miR-200b-001800 | 1.16 | 0.45 | 2.57 | 0.0344991 |
| hsa-miR-99b#-002196 | 1.04 | 0.5 | 2.06 | 0.034637 |
| hsa-miR-455-001280 | 0.19 | 0.92 | 0.2 | 0.0350124 |
| mmu-miR-126-3p-4395339 | 0.18 | 0.85 | 0.21 | 0.0353066 |
| mmu-miR-541-002562 | 6.35 | 0.15 | 42.21 | 0.0357332 |
| mmu-miR-434-5p-4395711 | 7.82 | 0.47 | 16.67 | 0.0381484 |
| mmu-miR-337-000193 | 11.58 | 0.44 | 26.6 | 0.0389707 |
| mmu-miR-410-4378093 | 11.25 | 0.29 | 39.01 | 0.0389754 |
| mmu-miR-328-4373049 | 0.44 | 1.66 | 0.26 | 0.0401884 |
| mmu-miR-136-4395641 | 9.92 | 0.26 | 37.87 | 0.0409448 |
| mmu-miR-379-4373349 | 12.61 | 0.42 | 30.2 | 0.041515 |
| mmu-miR-322#-002506 | 0.21 | 0.82 | 0.26 | 0.0418563 |
| mmu-miR-301a-4373064 | 0.39 | 1.02 | 0.38 | 0.0421615 |
| mmu-miR-411-4381013 | 8.82 | 0.3 | 29.89 | 0.0424654 |
| mmu-miR-125b-5p-4373148 | 0.34 | 2.25 | 0.15 | 0.043495 |
| mmu-miR-296-5p-4373066 | 0.44 | 1.71 | 0.26 | 0.0435887 |
| mmu-miR-326-001061 | 0.52 | 2.04 | 0.25 | 0.0448205 |
| mmu-miR-2146-241082_mat | 1.45 | 0.35 | 4.15 | 0.0463793 |
| mmu-miR-28#-002545 | 1.85 | 0.46 | 4.06 | 0.0470823 |
| mmu-miR-380-5p-4395731 | 7.62 | 0.4 | 18.91 | 0.0485808 |

Supplementary Table 4: miRNAs expression analysis in hyperplasia and non-neoplastic lung tissue derived from K-Ras^(+/LSLG12Vgeo);RERTn(ert/ert)^ mice after Tamoxifen administration. Hyp, hyperplasia; 4-OHT+N, non-neoplastic lung tissue after Tamoxifen administration.

| **UniqueID** | **Geom mean of intensities in class 1 Hyp** | **Geom mean of intensities in class 2 4-OHT+N** | **Fold-change (Hyp/4-OHT+N)** | **Parametric p-value** |
| --- | --- | --- | --- | --- |
| mmu-miR-370-4395386 | 1.81 | 0.00032 | 5598.12 | 9.14E-05 |
| mmu-miR-10a-4373153 | 0.31 | 1.44 | 0.22 | 0.0002117 |
| mmu-let-7a-4373169 | 2.60E-05 | 0.78 | 3.30E-05 | 0.0002731 |
| hsa-miR-93#-002139 | 1.12 | 0.51 | 2.21 | 0.0004927 |
| mmu-miR-674#-001956 | 1.44 | 0.21 | 7.02 | 0.0007543 |
| mmu-miR-339-5p-4395368 | 0.00045 | 1.11 | 0.00041 | 0.0007544 |
| mmu-miR-133b-4395358 | 0.00072 | 1.73 | 0.00042 | 0.000955 |
| mmu-miR-145-4395389 | 0.22 | 1.27 | 0.17 | 0.0009837 |
| mmu-miR-351-4373345 | 0.0017 | 1.97 | 0.00084 | 0.0013901 |
| mmu-miR-34b-3p-4395748 | 0.18 | 1.67 | 0.11 | 0.0021009 |
| mmu-miR-184-4373113 | 3.39 | 0.097 | 34.75 | 0.0022574 |
| mmu-miR-455-4395585 | 0.0079 | 2.35 | 0.0034 | 0.0029889 |
| mmu-miR-411-4381013 | 1.82 | 0.34 | 5.44 | 0.0031583 |
| mmu-miR-434-3p-4395734 | 1.69 | 0.24 | 6.88 | 0.0031899 |
| mmu-miR-146b-4373178 | 2.27 | 0.21 | 11.08 | 0.0033791 |
| hsa-miR-136#-002100 | 1.2 | 0.34 | 3.57 | 0.0034289 |
| hsa-miR-421-002700 | 1.47 | 0.42 | 3.51 | 0.0035772 |
| mmu-miR-181c-4373115 | 0.33 | 0.72 | 0.46 | 0.0038961 |
| hsa-miR-99b#-002196 | 1.67 | 0.46 | 3.6 | 0.0039337 |
| hsa-miR-23a#-002439 | 0.073 | 1.33 | 0.055 | 0.0039584 |
| mmu-miR-485-3p-001943 | 1.42 | 0.1 | 14.14 | 0.0039612 |
| mmu-miR-142-3p-4373136 | 0.27 | 1.24 | 0.22 | 0.0040946 |
| mmu-miR-34c-4373036 | 0.022 | 3.35 | 0.0066 | 0.0041636 |
| mmu-miR-34b-001065 | 0.022 | 1.68 | 0.013 | 0.0041819 |
| mmu-miR-29b#-002497 | 1.67 | 0.29 | 5.7 | 0.0046762 |
| mmu-miR-200c-4395411 | 1.52 | 0.34 | 4.45 | 0.004926 |
| mmu-miR-410-4378093 | 2.86 | 0.26 | 11.07 | 0.0060137 |
| mmu-miR-143-4395360 | 0.37 | 1.07 | 0.35 | 0.0062955 |
| hsa-miR-200c-000505 | 1.14 | 0.26 | 4.42 | 0.0090504 |
| mmu-miR-376a-4373347 | 2.29 | 0.17 | 13.8 | 0.009389 |
| mmu-miR-375-4373027 | 0.32 | 1.88 | 0.17 | 0.0097656 |
| mmu-miR-188-5p-4395431 | 1.44 | 0.42 | 3.43 | 0.0098505 |
| mmu-miR-1937b-241023_mat | 1.21 | 0.34 | 3.53 | 0.0117011 |
| mmu-miR-361-4373035 | 0.37 | 1.06 | 0.34 | 0.0126406 |
| mmu-miR-140-4373374 | 0.44 | 1.31 | 0.34 | 0.0131533 |
| mmu-miR-335-5p-4373045 | 0.33 | 1.21 | 0.27 | 0.0134699 |
| hsa-miR-136-000592 | 1.5 | 0.21 | 6.99 | 0.0147052 |
| hsa-miR-183#-002270 | 1.76 | 0.31 | 5.64 | 0.01524 |
| mmu-miR-467a-4395717 | 0.36 | 1.06 | 0.34 | 0.0153734 |
| mmu-miR-449b-001667 | 0.63 | 3.63 | 0.17 | 0.0160756 |
| mmu-miR-134-4373299 | 2.27 | 0.2 | 11.38 | 0.0166333 |
| mmu-miR-31#-002495 | 1.78 | 0.25 | 7.06 | 0.0170297 |
| mmu-miR-369-5p-4373195 | 2.02 | 0.058 | 34.94 | 0.017722 |
| mmu-miR-205-4373093 | 0.22 | 4.02 | 0.054 | 0.0181937 |
| rno-miR-1-4395765 | 0.029 | 1.71 | 0.017 | 0.0193093 |
| mmu-miR-182-4395729 | 1.62 | 0.6 | 2.72 | 0.0201955 |
| mmu-miR-126-5p-4373269 | 0.24 | 1.11 | 0.22 | 0.0220565 |
| mmu-miR-133a-4395357 | 0.12 | 1.35 | 0.087 | 0.0230221 |
| mmu-miR-541-002562 | 1.95 | 0.14 | 13.89 | 0.0253781 |
| mmu-miR-193#-002577 | 0.46 | 1.04 | 0.44 | 0.0283199 |
| mmu-miR-431-4395173 | 7.58 | 0.22 | 34.76 | 0.0291674 |
| mmu-miR-544-4395680 | 2.01 | 0.18 | 10.88 | 0.0320423 |
| mmu-miR-1-4395333 | 0.012 | 1.74 | 0.0067 | 0.0324274 |
| mmu-miR-409-3p-4395443 | 3.07 | 0.17 | 17.64 | 0.0329076 |
| mmu-miR-877#-002548 | 1.51 | 0.56 | 2.7 | 0.0336459 |
| mmu-miR-449a-4373207 | 0.5 | 2.89 | 0.17 | 0.0339905 |
| mmu-miR-674-4395193 | 1.3 | 0.58 | 2.24 | 0.0344505 |
| mmu-miR-137-4373301 | 0.15 | 1.38 | 0.11 | 0.0344596 |
| mmu-miR-491-4381053 | 1.27 | 0.58 | 2.2 | 0.036141 |
| mmu-miR-323-3p-4395338 | 9.67 | 0.22 | 44.31 | 0.0396017 |
| mmu-miR-542-3p-4378101 | 0.22 | 1.05 | 0.21 | 0.0397017 |
| mmu-miR-125a-5p-4395309 | 2.92 | 0.42 | 7.01 | 0.0401934 |
| mmu-miR-1940-121187_mat | 74.47 | 0.1 | 721.54 | 0.0403641 |
| mmu-miR-720-001629 | 1.2 | 0.54 | 2.21 | 0.0430022 |
| mmu-miR-99a-4373008 | 0.32 | 0.77 | 0.42 | 0.0445841 |
| mmu-miR-34b-5p-002617 | 0.036 | 2.14 | 0.017 | 0.0457778 |
| mmu-miR-183-4395380 | 1.3 | 0.3 | 4.29 | 0.0469047 |
| mmu-miR-682-4381081 | 0.49 | 1.12 | 0.43 | 0.0478441 |
| mmu-miR-1188-002866 | 1.48 | 0.4 | 3.73 | 0.048565 |
| mmu-miR-27a-4373287 | 0.23 | 1.35 | 0.17 | 0.0488028 |

Supplementary Table 5: miRNAs expression analysis in adenoma and non-neoplastic lung tissue derived from K-Ras^(+/LSLG12Vgeo);RERTn(ert/ert)^ transgenic mice after Tamoxifen administration. Ad, adenoma; 4-OHT+N, normal lung tissue after Tamoxifen administration.

| **UniqueID** | **Geom mean of intensities in class 1 Ad** | **Geom mean of intensities in class 2 4-OHT+N** | **Fold-change (Ad/4-OHT+N)** | **Parametric p-value** |
| --- | --- | --- | --- | --- |
| mmu-miR-674#-001956 | 1.87 | 0.21 | 9.09 | 0.0003351 |
| mmu-miR-489-4378114 | 0.078 | 0.6 | 0.13 | 0.0006512 |
| mmu-miR-370-4395386 | 7.04 | 0.00032 | 21786.29 | 0.0006613 |
| mmu-miR-376a#-002482 | 279.42 | 0.087 | 3218.23 | 0.0006697 |
| mmu-miR-142-5p-4395359 | 0.12 | 0.92 | 0.13 | 0.0007629 |
| mmu-miR-351-4373345 | 5.00E-04 | 1.97 | 0.00025 | 0.0013112 |
| mmu-miR-322#-002506 | 0.21 | 0.77 | 0.28 | 0.0015522 |
| mmu-miR-199b-001131 | 0.19 | 0.69 | 0.28 | 0.0016611 |
| mmu-miR-543-001298 | 21.42 | 0.019 | 1149.23 | 0.0017887 |
| mmu-miR-455-4395585 | 0.0024 | 2.35 | 0.001 | 0.0020021 |
| hsa-miR-144-002676 | 8.00E-04 | 0.93 | 0.00087 | 0.0020675 |
| hsa-miR-338-000548 | 0.012 | 0.67 | 0.018 | 0.0021763 |
| mmu-miR-10a-4373153 | 0.3 | 1.44 | 0.21 | 0.002303 |
| mmu-miR-126-5p-4373269 | 0.23 | 1.11 | 0.21 | 0.0025303 |
| mmu-miR-335-5p-4373045 | 0.1 | 1.21 | 0.083 | 0.0026678 |
| mmu-miR-184-4373113 | 3.28 | 0.097 | 33.71 | 0.0032687 |
| mmu-miR-133a-4395357 | 0.045 | 1.35 | 0.034 | 0.003512 |
| mmu-miR-34b-3p-4395748 | 0.098 | 1.67 | 0.059 | 0.0037632 |
| mmu-miR-878-3p-002541 | 0.0024 | 1.09 | 0.0022 | 0.0049738 |
| mmu-miR-31#-002495 | 2.91 | 0.25 | 11.52 | 0.0051342 |
| hsa-miR-9#-002231 | 0.025 | 0.87 | 0.028 | 0.005243 |
| hsa-miR-200b-001800 | 1.16 | 0.53 | 2.2 | 0.005487 |
| mmu-miR-451-4373360 | 0.082 | 1.99 | 0.041 | 0.006075 |
| hsa-miR-93#-002139 | 1.14 | 0.51 | 2.24 | 0.006368 |
| mmu-miR-145-4395389 | 0.21 | 1.27 | 0.17 | 0.0067996 |
| mmu-miR-293-4386754 | 0.022 | 0.87 | 0.025 | 0.007704 |
| hsa-miR-99b#-002196 | 1.04 | 0.46 | 2.24 | 0.007726 |
| mmu-miR-450B-3P-002632 | 0.23 | 0.58 | 0.4 | 0.0079511 |
| mmu-miR-142-3p-4373136 | 0.23 | 1.24 | 0.19 | 0.0081922 |
| mmu-miR-493-4395649 | 92.75 | 0.38 | 242.06 | 0.0083138 |
| mmu-miR-384-5p-4395732 | 0.012 | 1.44 | 0.008 | 0.0084079 |
| mmu-miR-31-4373331 | 3.12 | 0.41 | 7.53 | 0.0098927 |
| mmu-miR-200c-4395411 | 1.64 | 0.34 | 4.78 | 0.0107008 |
| mmu-miR-485-3p-001943 | 3.64 | 0.1 | 36.15 | 0.011408 |
| mmu-miR-199a-3p-4395415 | 0.37 | 0.9 | 0.42 | 0.0120133 |
| mmu-miR-146b-4373178 | 1.73 | 0.21 | 8.43 | 0.0127455 |
| mmu-miR-335-3p-4395296 | 0.12 | 0.67 | 0.18 | 0.0129861 |
| mmu-miR-195-4373105 | 0.38 | 0.91 | 0.42 | 0.0130723 |
| hsa-miR-200c-000505 | 1.46 | 0.26 | 5.68 | 0.01324 |
| mmu-miR-193-4395361 | 0.37 | 0.91 | 0.41 | 0.0142128 |
| mmu-miR-449a-4373207 | 0.27 | 2.89 | 0.094 | 0.0142201 |
| mmu-miR-409-3p-4395443 | 30.17 | 0.17 | 173.1 | 0.018021 |
| mmu-miR-487b-001306 | 4.06 | 0.36 | 11.28 | 0.0181963 |
| mmu-miR-376a-4373347 | 7.95 | 0.17 | 47.92 | 0.0194806 |
| mmu-miR-434-3p-4395734 | 7.43 | 0.24 | 30.34 | 0.0217805 |
| mmu-miR-380-5p-4395731 | 7.62 | 0.45 | 16.91 | 0.0219948 |
| mmu-miR-27a-4373287 | 0.4 | 1.35 | 0.3 | 0.0220006 |
| mmu-miR-143-4395360 | 0.25 | 1.07 | 0.24 | 0.0240493 |
| mmu-miR-1-4395333 | 0.018 | 1.74 | 0.01 | 0.0245024 |
| mmu-miR-1944-121189_mat | 1.43 | 0.41 | 3.53 | 0.0252722 |
| mmu-miR-34c#-002584 | 0.072 | 2.09 | 0.035 | 0.0270707 |
| mmu-miR-28#-002545 | 1.85 | 0.53 | 3.52 | 0.0271535 |
| mmu-miR-449b-001667 | 0.25 | 3.63 | 0.07 | 0.0271892 |
| mmu-miR-205-4373093 | 0.15 | 4.02 | 0.037 | 0.0290736 |
| mmu-miR-434-5p-4395711 | 7.82 | 0.42 | 18.6 | 0.0306418 |
| mmu-miR-181a-4373117 | 0.31 | 0.85 | 0.37 | 0.0306662 |
| mmu-miR-365-4373194 | 0.17 | 1.41 | 0.12 | 0.0312841 |
| mmu-miR-140-4373374 | 0.48 | 1.31 | 0.37 | 0.0322037 |
| mmu-miR-497-4381046 | 0.39 | 1.06 | 0.37 | 0.032819 |
| mmu-miR-150-4373127 | 0.15 | 0.66 | 0.23 | 0.0336691 |
| mmu-miR-134-4373299 | 13.86 | 0.2 | 69.58 | 0.0339131 |
| mmu-miR-126-3p-4395339 | 0.18 | 0.77 | 0.24 | 0.0346611 |
| mmu-miR-410-4378093 | 11.25 | 0.26 | 43.48 | 0.035325 |
| mmu-miR-1941-5p-121140_mat | 0.058 | 1.46 | 0.039 | 0.0377248 |
| mmu-miR-326-001061 | 0.52 | 1.49 | 0.35 | 0.0407 |
| mmu-miR-337-000193 | 11.58 | 0.4 | 28.75 | 0.0408024 |
| mmu-miR-338-3p-4395363 | 0.035 | 1.08 | 0.033 | 0.043176 |
| mmu-miR-345-5p-4395658 | 1.45 | 0.6 | 2.42 | 0.0435347 |
| hsa-miR-136-000592 | 5.76 | 0.21 | 26.89 | 0.0438197 |
| mmu-miR-136#-002512 | 3.76 | 0.41 | 9.19 | 0.0453842 |
| mmu-miR-379-4373349 | 12.61 | 0.38 | 33.45 | 0.0469574 |
| mmu-miR-136-4395641 | 9.92 | 0.3 | 33.2 | 0.0478775 |
| mmu-miR-376b#-002451 | 7.57 | 0.33 | 22.76 | 0.0479902 |
| mmu-miR-411-4381013 | 8.82 | 0.34 | 26.33 | 0.0481193 |
| mmu-miR-182-4395729 | 1.39 | 0.6 | 2.33 | 0.0496256 |
| mmu-miR-544-4395680 | 9.94 | 0.18 | 53.77 | 0.0496453 |

**Supplementary Table 6**: miRNAs expression analysis in adenoma and hyperplasia derived from K-Ras^(+/LSLG12Vgeo);RERTn(ert/ert)^ mice. Ad, adenoma; Hyp, hyperplasia.

| **UniqueID** | **Geom mean of intensities in class 1 Ad** | **Geom mean of intensities in class 2 Hyp** | **Fold-change (Ad/Hyp)** | **Parametric p-value** |
| --- | --- | --- | --- | --- |
| mmu-miR-142-5p-4395359 | 0.12 | 0.5 | 0.24 | 3.64E-05 |
| hsa-miR-338-000548 | 0.012 | 0.59 | 0.02 | 0.0089647 |
| hsa-miR-23a#-002439 | 1.85 | 0.073 | 25.38 | 0.0178382 |
| mmu-miR-384-5p-4395732 | 0.012 | 0.39 | 0.029 | 0.0197267 |
| mmu-miR-2138-241080_mat | 3.74 | 1.01 | 3.7 | 0.0234996 |
| mmu-miR-335-5p-4373045 | 0.1 | 0.33 | 0.31 | 0.0282433 |
| mmu-miR-682-4381081 | 2.11 | 0.49 | 4.34 | 0.0300977 |
| mmu-miR-491-4381053 | 0.48 | 1.27 | 0.38 | 0.0446426 |
| mmu-miR-146a-4373132 | 0.36 | 0.93 | 0.38 | 0.0466845 |

Supplementary Table 7: a-CGH analysis of chr.14 was performed in 20 lung tumors and genomic derangements were detected for five cases (Thresholds: log_2_ < -1 = deletion; -1 ≤ log_2_ < 0 = loss; 0 < log_2_ < 2 = gain; log_2_ ≥ 2 = amplification).

| **Sample** | **Chr** | **Cytoband** | **Start** | **Stop** | **Mb** | **Gain**  **(log_2_ Value)** | **Loss**  **(log_2_ Value)** |
| --- | --- | --- | --- | --- | --- | --- | --- |
| 13 | chr14 | q24.1 - q32.33 | 69,341,338 | 107,258,824 | 38 | 0 | -0.29 |
| 15 | chr14 | q11.2 - q32.33 | 20,253,739 | 107,258,824 | 87 | 0.25 | 0 |
| 17 | chr14 | q32.32 - q32.33 | 103,474,857 | 104,263,828 | 0.8 | 1.22 | 0 |
| 18 | chr14 | q31.3 - q32.32 | 89,172,615 | 103,447,263 | 14 | 0.45 | 0 |
| 19 | chr14 | q21.3 - q32.33 | 49,914,341 | 107,258,824 | 57 | 0.19 | 0 |

Supplementary Table 8: Gene expression assays list (Thermo Fisher Scientific).

| **Entrez Gene** | **Gene Symbol** | **Gene Name** | **Assay ID** |
| --- | --- | --- | --- |
| [9429](http://www.ncbi.nlm.nih.gov/entrez/query.fcgi?db=gene&cmd=Retrieve&dopt=full_report&list_uids=9429) | *ABCG2* | ATP-Binding Cassette, Sub-Family G (WHITE), Member 2 | Hs01053790_m1 |
| 216 | *ALDH1A1* | Aldehyde Dehydrogenase 1 Family, Member A1 | Hs00946916_m1 |
| [581](http://www.ncbi.nlm.nih.gov/entrez/query.fcgi?db=gene&cmd=Retrieve&dopt=full_report&list_uids=581) | *BAX* | BCL2-Associated X Protein | Hs00180269_m1 |
| [598](http://www.ncbi.nlm.nih.gov/entrez/query.fcgi?db=gene&cmd=Retrieve&dopt=full_report&list_uids=598) | *BCL2L1* | BCL2-Like 1 | Hs00608023_m1 |
| [637](http://www.ncbi.nlm.nih.gov/entrez/query.fcgi?db=gene&cmd=Retrieve&dopt=full_report&list_uids=637) | *BID* | BH3 Interacting Domain Death Agonist | Hs00609630_m1 |
| [638](http://www.ncbi.nlm.nih.gov/entrez/query.fcgi?db=gene&cmd=Retrieve&dopt=full_report&list_uids=638) | *BIK* | BCL2-Interacting Killer | Hs00609635_m1 |
| [332](http://www.ncbi.nlm.nih.gov/entrez/query.fcgi?db=gene&cmd=Retrieve&dopt=full_report&list_uids=332) | *BIRC5* | Baculoviral IAP Repeat Containing 5 | Hs00153353_m1 |
| [960](http://www.ncbi.nlm.nih.gov/entrez/query.fcgi?db=gene&cmd=Retrieve&dopt=full_report&list_uids=960) | *CD44* | CD44 Molecule | Hs01075861_m1 |
| [1026](http://www.ncbi.nlm.nih.gov/entrez/query.fcgi?db=gene&cmd=Retrieve&dopt=full_report&list_uids=1026) | *CDKN1A* | Cyclin-Dependent Kinase Inhibitor 1A | Hs00355782_m1 |
| 4609 | *cMYC* | V-Myc Avian Myelocytomatosis Viral Oncogene Homolog | Hs00153408_m1 |
| [92359](http://www.ncbi.nlm.nih.gov/entrez/query.fcgi?db=gene&cmd=Retrieve&dopt=full_report&list_uids=92359) | *CRB3* | Crumbs Family Member 3 | Hs00373616_m1 |
| [1601](http://www.ncbi.nlm.nih.gov/entrez/query.fcgi?db=gene&cmd=Retrieve&dopt=full_report&list_uids=1601) | *DAB2* | Dab, Mitogen-Responsive Phosphoprotein, Homolog 2 | Hs00184598_m1 |
| [22943](http://www.ncbi.nlm.nih.gov/entrez/query.fcgi?db=gene&cmd=Retrieve&dopt=full_report&list_uids=22943) | *DKK1* | Dickkopf WNT Signaling Pathway Inhibitor 1 | Hs00183740_m1 |
| [3280](http://www.ncbi.nlm.nih.gov/entrez/query.fcgi?db=gene&cmd=Retrieve&dopt=full_report&list_uids=3280) | *HES1* | Hes Family BHLH Transcription Factor 1 | Hs00172878_m1 |
| [3159](http://www.ncbi.nlm.nih.gov/entrez/query.fcgi?db=gene&cmd=Retrieve&dopt=full_report&list_uids=3159) | *HMGA1* | High Mobility Group AT-Hook 1 | Hs00852949_g1 |
| [8091](http://www.ncbi.nlm.nih.gov/entrez/query.fcgi?db=gene&cmd=Retrieve&dopt=full_report&list_uids=8091) | *HMGA2* | High Mobility Group AT-Hook 2 | Hs00171869_m1 |
| [9314](http://www.ncbi.nlm.nih.gov/entrez/query.fcgi?db=gene&cmd=Retrieve&dopt=full_report&list_uids=9314) | *KLF4* | Kruppel-Like Factor 4 | Hs00358836_m1 |
| 79923 | *NANOG* | **Nanog** Homeobox | Hs04260366_g1 |
| 4170 | *MCL1* | Myeloid Cell Leukemia 1 | Hs01050896_m1 |
| [8650](http://www.ncbi.nlm.nih.gov/entrez/query.fcgi?db=gene&cmd=Retrieve&dopt=full_report&list_uids=8650) | *NUMB* | Numb Homolog (Drosophila) | Hs01105433_m1 |
| [9253](http://www.ncbi.nlm.nih.gov/entrez/query.fcgi?db=gene&cmd=Retrieve&dopt=full_report&list_uids=9253) | *NUMBL* | Numb Homolog (Drosophila)-Like | Hs00191080_m1 |
| [7157](http://www.ncbi.nlm.nih.gov/entrez/query.fcgi?db=gene&cmd=Retrieve&dopt=full_report&list_uids=7157) | *P53* | Tumor Protein P53 | Hs00153340_m1 |
| [8626](http://www.ncbi.nlm.nih.gov/entrez/query.fcgi?db=gene&cmd=Retrieve&dopt=full_report&list_uids=8626) | *P63* | Tumor Protein P63 | Hs00186613_m1 |
| [7161](http://www.ncbi.nlm.nih.gov/entrez/query.fcgi?db=gene&cmd=Retrieve&dopt=full_report&list_uids=7161) | *P73* | Tumor Protein P73 | Hs00232088_m1 |
| [5111](http://www.ncbi.nlm.nih.gov/entrez/query.fcgi?db=gene&cmd=Retrieve&dopt=full_report&list_uids=5111) | *PCNA* | Proliferating Cell Nuclear Antigen | Hs00696862_m1 |
| [8842](http://www.ncbi.nlm.nih.gov/entrez/query.fcgi?db=gene&cmd=Retrieve&dopt=full_report&list_uids=8842) | *PROM1* | CD133 | Hs01009250_m1 |
| [5728](http://www.ncbi.nlm.nih.gov/entrez/query.fcgi?db=gene&cmd=Retrieve&dopt=full_report&list_uids=5728) | *PTEN* | Phosphatase And Tensin Homolog | Hs02621230_s1 |
| [3516](http://www.ncbi.nlm.nih.gov/entrez/query.fcgi?db=gene&cmd=Retrieve&dopt=full_report&list_uids=3516) | *RBP-jK* | Recombination Signal Binding Protein For Immunoglobulin Kappa J Region | Hs01068138_m1 |
| [6271](http://www.ncbi.nlm.nih.gov/entrez/query.fcgi?db=gene&cmd=Retrieve&dopt=full_report&list_uids=6271) | *S100A* | S100 Calcium Binding Protein A1 | Hs00195582_m1 |
| [10110](http://www.ncbi.nlm.nih.gov/entrez/query.fcgi?db=gene&cmd=Retrieve&dopt=full_report&list_uids=10110) | *SGK2* | Serum/Glucocorticoid Regulated Kinase 2 | Hs00367639_m1 |
| [6615](http://www.ncbi.nlm.nih.gov/entrez/query.fcgi?db=gene&cmd=Retrieve&dopt=full_report&list_uids=6615) | *SNAI1* | Snail Family Zinc Finger 1 | Hs00195591_m1 |
| [6591](http://www.ncbi.nlm.nih.gov/entrez/query.fcgi?db=gene&cmd=Retrieve&dopt=full_report&list_uids=6591) | *SNAI2* | Snail Family Zinc Finger 2 | Hs00161904_m1 |
| 6657 | *SOX2* | SRY(Sex Determining RegionY)-Box 2 | Hs01053049_s1 |
| [6678](http://www.ncbi.nlm.nih.gov/entrez/query.fcgi?db=gene&cmd=Retrieve&dopt=full_report&list_uids=6678) | *SPARC* | Secreted Protein, Acidic, Cysteine-Rich | Hs00234160_m1 |
| [7291](http://www.ncbi.nlm.nih.gov/entrez/query.fcgi?db=gene&cmd=Retrieve&dopt=full_report&list_uids=7291) | *TWIST* | Twist Family BHLH Transcription Factor 1 | Hs00361186_m1 |
| [7431](http://www.ncbi.nlm.nih.gov/entrez/query.fcgi?db=gene&cmd=Retrieve&dopt=full_report&list_uids=7431) | *VIM* | Vimentin | Hs00185584_m1 |
| [6935](http://www.ncbi.nlm.nih.gov/entrez/query.fcgi?db=gene&cmd=Retrieve&dopt=full_report&list_uids=6935) | *ZEB1* | Zinc Finger E-Box Binding Homeobox 1 | Hs00232783_m1 |
| [9839](http://www.ncbi.nlm.nih.gov/entrez/query.fcgi?db=gene&cmd=Retrieve&dopt=full_report&list_uids=9839) | *ZEB2* | Zinc Finger E-Box Binding Homeobox 2 | Hs00207691_m1 |

Supplementary Table 9: Cancer 10-Pathway Reporter Array Cignal Finder Reporter Array (SaBiosciences Corp.)

| **Pathway** | **Transcription Factor** |
| --- | --- |
| Wnt | TCF/LEF |
| Notch | RBP-Jk |
| p53/DNA Damage | p53 |
| TGFβ | SMAD2/3/4 |
| Cell Cycle/pRB-E2F | E2F/DP1 |
| NFkB | NFkB |
| Myc/Max | Myc/Max |
| Hypoxia | HIF1A |
| MAPK/ERK | Elk-1/SRF |
| MAPK/JNK | AP-1 |

**Supplementary Table 10**: K-Ras^(+/LSLG12Vgeo);RERTn(ert/ert)^ mice used for lung tissues and lesions (Hyp, Ad and AdCa) isolation by laser assisted microdissection.

| **Mouse** | **Oncogene induction (Yes/No)** | **Age (Months)** | **Sex (M/F)** | **Isolated samples** |
| --- | --- | --- | --- | --- |
| 1 | N | 9 | M | Normal Lung Tissue (N) |
| 2 | N | 9 | M | Normal Lung Tissue (N) |
| 3 | N | 9 | M | Normal Lung Tissue (N) |
| 4 | N | 9 | F | Normal Lung Tissue (N) |
| 5 | Y | 9 | F | Non Neoplastic Lung Tissue (4-OHT N) |
|  |  |  |  | Hyperplesia (Hyp) |
|  |  |  |  | Adenoma (Ad) |
|  |  |  |  | Adenocarcinoma (AdCa) |
| 6 | Y | 9 | F | Non Neoplastic Lung Tissue (4-OHT N) |
|  |  |  |  | Hyperplesia (Hyp) |
|  |  |  |  | Adenoma (Ad) |
|  |  |  |  | Adenocarcinoma (AdCa) |
| 7 | Y | 9 | M | Non Neoplastic Lung Tissue (4-OHT N) |
|  |  |  |  | Hyperplesia (Hyp) |
|  |  |  |  | Adenoma (Ad) |
|  |  |  |  | Adenocarcinoma (AdCa) |
| 8 | Y | 9 | M | Non Neoplastic Lung Tissue (4-OHT N) |
|  |  |  |  | Hyperplesia (Hyp) |
|  |  |  |  | Adenoma (Ad) |
|  |  |  |  | Adenocarcinoma (AdCa) |
| 9 | N | 5 | M | Normal Lung Tissue (N) |
| 10 | N | 5 | M | Normal Lung Tissue (N) |
| 11 | Y | 5 | M | Non Neoplastic Lung Tissue (4-OHT N) |
|  |  |  |  | Hyperplesia (Hyp) |
|  |  |  |  | Adenoma (Ad) |
| 12 | Y | 5 | M | Non Neoplastic Lung Tissue (4-OHT N) |
|  |  |  |  | Hyperplesia (Hyp) |
|  |  |  |  | Adenoma (Ad) |
| 12 | N | 2 | M | Normal Lung Tissue (N) |
| 13 | N | 2 | M | Normal Lung Tissue (N) |
| 14 | Y | 2 | M | Non Neoplastic Lung Tissue (4-OHT N) |
|  |  |  |  | Hyperplesia (Hyp) |
| 15 | Y | 2 | M | Non Neoplastic Lung Tissue (4-OHT N) |
|  |  |  |  | Hyperplesia (Hyp) |

Supplementary Table 11: Clinicopathological features of analyzed NSCLCs (n=113). Histological type (ADC, Adenocarcinoma; SCC, Squamous Cell Carcinoma; LCC, Large Cell Carcinoma), tumor grading and staging according with TNM system are indicated.

| **Case** | **Gender** | **Age** | **Histological Type** | **Grading** | **TNM Staging** |
| --- | --- | --- | --- | --- | --- |
| 1 | M | 73 | ADC | 3 | T2N1M0 |
| 2 | F | 56 | ADC | 2 | T2N0M0 |
| 3 | M | 64 | ADC/SCC | 2 | T2N0M0 |
| 4 | M | 59 | ADC | 3 | T2N0M0 |
| 5 | F | 57 | ADC | 1 | T2N0M0 |
| 6 | M | 57 | ADC/SCC | 1 | T2N2M0 |
| 7 | M | 73 | ADC | 3 | T2N1M0 |
| 8 | M | 56 | ADC | 3 | T3N0M0 |
| 9 | M | 69 | SCC | 2 | T2N0M0 |
| 10 | M | 63 | ADC | 2 | T2N2M0 |
| 11 | F | 71 | ADC | 2 | T2N1M0 |
| 12 | M | 78 | ADC | 3 | T4N0M1 |
| 13 | F | 51 | SCC | 3 | T1N0M0 |
| 14 | M | 71 | SCC | 3 | T2N0M0 |
| 15 | F | 59 | ADC | 2 | T1N1M0 |
| 16 | M | 60 | ADC | 2 | T2N0M0 |
| 17 | M | 64 | ADC | 3 | T2N0M0 |
| 18 | M | 71 | SCC | 2 | T4N0M0 |
| 19 | M | 77 | ADC | 2 | T2N0M0 |
| 20 | M | 75 | SCC | 2 | T1N0M0 |
| 21 | M | 67 | ADC | 2 | T1N0M0 |
| 22 | M | 68 | ADC | 2 | T2N0M0 |
| 23 | M | 64 | ADC | 3 | T2N0M0 |
| 24 | F | 69 | ADC | 2 | T2N1M0 |
| 25 | M | 63 | ADC | 2 | T3N2M0 |
| 26 | M | 72 | SCC | 3 | T2N2M0 |
| 27 | M | 66 | ADC | 3 | T4N2M0 |
| 28 | F | 62 | ADC | 3 | T4N2M0 |
| 29 | M | 59 | ADC | 1 | T4N0M0 |
| 30 | M | 65 | ADC | 3 | T3N2M0 |
| 31 | F | 50 | ADC | 2 | T2N0M0 |
| 32 | M | 51 | SCC | 3 | T2N0M0 |
| 33 | F | 66 | ADC | 2 | T2N2M0 |
| 34 | M | 73 | SCC | 2 | T2N2M0 |
| 35 | M | 55 | SCC | 3 | T2N1M0 |
| 36 | M | 70 | SCC | 2 | T1N0M0 |
| 37 | M | 71 | SCC | 3 | T2N0M0 |
| 38 | F | 67 | ADC | 3 | T1N0M0 |
| 39 | M | 75 | SCC | 3 | T1N1M0 |
| 40 | M | 55 | ADC | 3 | T2N2M0 |
| 41 | M | 72 | ADC | 3 | T2N1M0 |
| 42 | M | 69 | ADC | 3 | T4N2M0 |
| 43 | M | 67 | ADC | 3 | T2N1M1 |
| 44 | F | 58 | ADC | 1 | T1N0M0 |
| 45 | M | 74 | ADC | 2 | T1N0M0 |
| 46 | M | 54 | ADC | 3 | T2N0M0 |
| 47 | M | 54 | ADC | 2 | T2N0M0 |
| 48 | M | 60 | ADC | 2 | T2N2M0 |
| 49 | M | 56 | ADC | 2 | T2N1M0 |
| 50 | M | 78 | SCC | 3 | T1N0M0 |
| 51 | M | 70 | SCC | 2 | T2N0M0 |
| 52 | F | 71 | ADC | 2 | T4N2M0 |
| 53 | M | 75 | ADC | 2 | T2N0M0 |
| 54 | M | 80 | ADC | 1 | T2N0M0 |
| 55 | M | 72 | ADC | 3 | T2N0M0 |
| 56 | M | 65 | SCC | 3 | T2N1M0 |
| 57 | M | 73 | ADC | 3 | T2N1M0 |
| 58 | F | 56 | ADC | 2 | T2N0M0 |
| 59 | M | 64 | ADC | 3 | T2N0M0 |
| 60 | M | 61 | ADC | 3 | T2N0M0 |
| 61 | F | 81 | ADC | 2 | T2N1M0 |
| 62 | F | 59 | ADC | 2 | T3N3M0 |
| 63 | M | 77 | ADC | 3 | T2N1M0 |
| 64 | M | 68 | LCC | 3 | T2N1M0 |
| 65 | M | 44 | ADC | 2 | T2N1M0 |
| 66 | M | 71 | ADC | 3 | T1N0M0 |
| 67 | M | 58 | ADC | 2 | T1N0M0 |
| 68 | M | 67 | ADC | 3 | T2N2M0 |
| 69 | M | 63 | SCC | 3 | T3N1M0 |
| 70 | F | 57 | ADC | 3 | T2N1M0 |
| 71 | F | 71 | SCC | 2 | T2N0M0 |
| 72 | M | 58 | ADC | 2 | T4N1M0 |
| 73 | F | 74 | ADC | 2 | T2N0M0 |
| 74 | M | 62 | ADC | 2 | T2N0M0 |
| 75 | F | 50 | ADC | 3 | T2N1M0 |
| 76 | M | 60 | ADC | 1 | T1N0M0 |
| 77 | M | 67 | SCC | 1 | T2N2M0 |
| 78 | M | 54 | ADC | 2 | T2N1M0 |
| 79 | M | 76 | ADC | 2 | T2N2M0 |
| 80 | M | 67 | ADC | 3 | T3N1M0 |
| 81 | F | 44 | ADC | 2 | T1N0M0 |
| 82 | M | 73 | ADC/SCC | 2 | T1N2M0 |
| 83 | M | 61 | ADC | 2 | T2N0M0 |
| 84 | F | 60 | ADC | 2 | T2N2M0 |
| 85 | M | 77 | ADC | 3 | T2N0M0 |
| 86 | M | 71 | ADC | 3 | T2N2M0 |
| 87 | F | 64 | SCC | 2 | T1N0M0 |
| 88 | M | 68 | ADC | 2 | T2N0M0 |
| 89 | M | 73 | ADC | 2 | T1N0M0 |
| 90 | M | 53 | ADC | 3 | T1N0M0 |
| 91 | M | 71 | ADC | 2 | T1N2M0 |
| 92 | M | 70 | SCC | 3 | T1N0M0 |
| 93 | M | 75 | SCC | 3 | T2N0M0 |
| 94 | M | 53 | ADC | 3 | T2N1M0 |
| 95 | M | 72 | LCC | 3 | T3N1M0 |
| 96 | F | 69 | ADC | 2 | T2N0M0 |
| 97 | F | 68 | SCC | 3 | T3N0M0 |
| 98 | M | 72 | SCC | 2 | T2N0M0 |
| 99 | M | 75 | ADC | 2 | T1N2M0 |
| 100 | M | 77 | SCC | 3 | T2N1M0 |
| 101 | F | 59 | ADC | 2 | T3N2M0 |
| 102 | M | 71 | SCC | 3 | T2N0M0 |
| 103 | M | 70 | SCC | 2 | T2N0M0 |
| 104 | M | 68 | SCC | 2 | T1N0M0 |
| 105 | M | 54 | ADC | 3 | T2N0M0 |
| 106 | F | 69 | ADC | 2 | T2N2M0 |
| 107 | M | 59 | ADC | 2 | T1N2M0 |
| 108 | F | 57 | LCC | 3 | T2N0M0 |
| 109 | F | 61 | ADC | 2 | T2N0M0 |
| 110 | M | 68 | SCC | 2 | T1N0M0 |
| 111 | M | 73 | ADC | 3 | T3N2M0 |
| 112 | M | 69 | ADC | 1 | T1N0M0 |
| 113 | M | 61 | ADC | 3 | T1N0M0 |

Supplementary Table 12: miRNAs expression assay list (Thermo Fisher Scientific)

| **miRNA** | **Assay ID** |
| --- | --- |
| miR-127-3p | 000452 |
| miR-300-3p | 241035 |
| miR-370-3p | 002275 |
| miR-379-5p | 001138 |
| miR-382-5p | 000572 |
| miR-409-3p | 002332 |
| miR-412-3p | 001023 |
| miR-431-5p | 001979 |
| miR-494-3p | 002365 |
| miR-543-3p | 002376 |
